# Slight changes in the chemical structure of haemanthamine greatly influence the effect of the derivatives on rumen fermentation *in vitro*

**DOI:** 10.1038/s41598-019-38977-x

**Published:** 2019-02-21

**Authors:** Eva Ramos-Morales, Jamie Tibble-Howlings, Laura Lyons, Magnus O. Ogbu, Patrick J. Murphy, Radek Braganca, Charles James Newbold

**Affiliations:** 1 Scotland’s Rural College, Edinburgh, EH9 3JG UK; 2 Biocomposites Centre, Bangor University, Bangor, LL57 2UW UK; 3 Institute of Biological, Environmental and Rural Sciences, Aberystwyth University, SY23 3DA Aberystwyth, UK

## Abstract

Although the potential of plants extracts to improve feed efficiency and animal productivity and decrease methane emissions by enteric fermentation has been shown, the information available is often contradictory which has been attributed to differences in the complex mixture of bioactive compounds and their interactions. Understanding the degree to which structural features in a compound may affect the biological activity of an extract is essential. We hypothesised that relative small variations in the structure of a compound can have a significant influence on the ability of the derivatives to alter fermentation in the rumen. Nine compounds were synthetized from the natural alkaloid haemanthamine and tested *in vitro* for their effects on rumen protozoa and fermentation parameters. Our results showed that simple esterifications of haemanthamine or its derivative dihydrohaemanthamine with acetate, butyrate, pivalate or hexanoate led to compounds that differed in their effects on rumen fermentation.

## Introduction

Since the ban of antibiotics as growth promoting feed additives by the European Union in 2006, plant extracts and plant secondary metabolites (e.g. saponins, tannins and essential oils) have been widely investigated as alternatives to manipulate rumen fermentation^1^. Although the potential of plants extracts to increase productivity and decrease methane emissions has been shown^2^, the information available is often contradictory with apparently similar products having different biological effects^3^ (ie. saponin extracts/compounds differing in their ability to modulate fermentation *in vitro*^3,4^). This has been attributed to differences in the complex mixture of bioactive compounds and their interactions^5^. The composition of an extract can greatly vary according to the nature of the starting plant material (plant variety, harvest time, soil composition, altitude, climate, and processing and storage conditions) and the extraction and purification processes applied^3,6^. Indeed, large differences in composition between batches have been reported with saponin extracts even when prepared from the same substrate with the same methodology^3^. While standardized methods to ensure the homogeneity of plant extracts are needed, understanding the degree to which structural features in a compound may affect the biological activity of an extract is essential. Our hypothesis is that relative small variations in the structure of a compound can have a significant influence on the ability of such compounds to alter fermentation in the rumen.

Few studies on the structure-activity relationship that underly the mechanisms of action of pure compounds in the rumen have been published. Early studies by Bush *et al*.^7^ with derivatives of perloline, one of the major alkaloids in tall fescue, showed that the effect on rumen fermentation was greatly influenced by the substitution at the C-5 position. We have recently revisited this concept, showing that modifications in the structure of Hederoside B, the major saponin present in ivy fruit extract, resulted in saponin-like analogues with different biological activities in terms of antiprotozoal effect and stability of the molecules in the rumen^4^. In the present work we attempt to expand the concept that relative small variations in the structure of a compound can have a significant influence on their biological activity by using the natural alkaloid haemanthamine to study the effect of simple esterifications of this molecule and its derivative dihydrohaemanthamine on both rumen protozoa and rumen fermentation pattern.

## Results

### Haemanthamine derivatives

**Figure 1:**
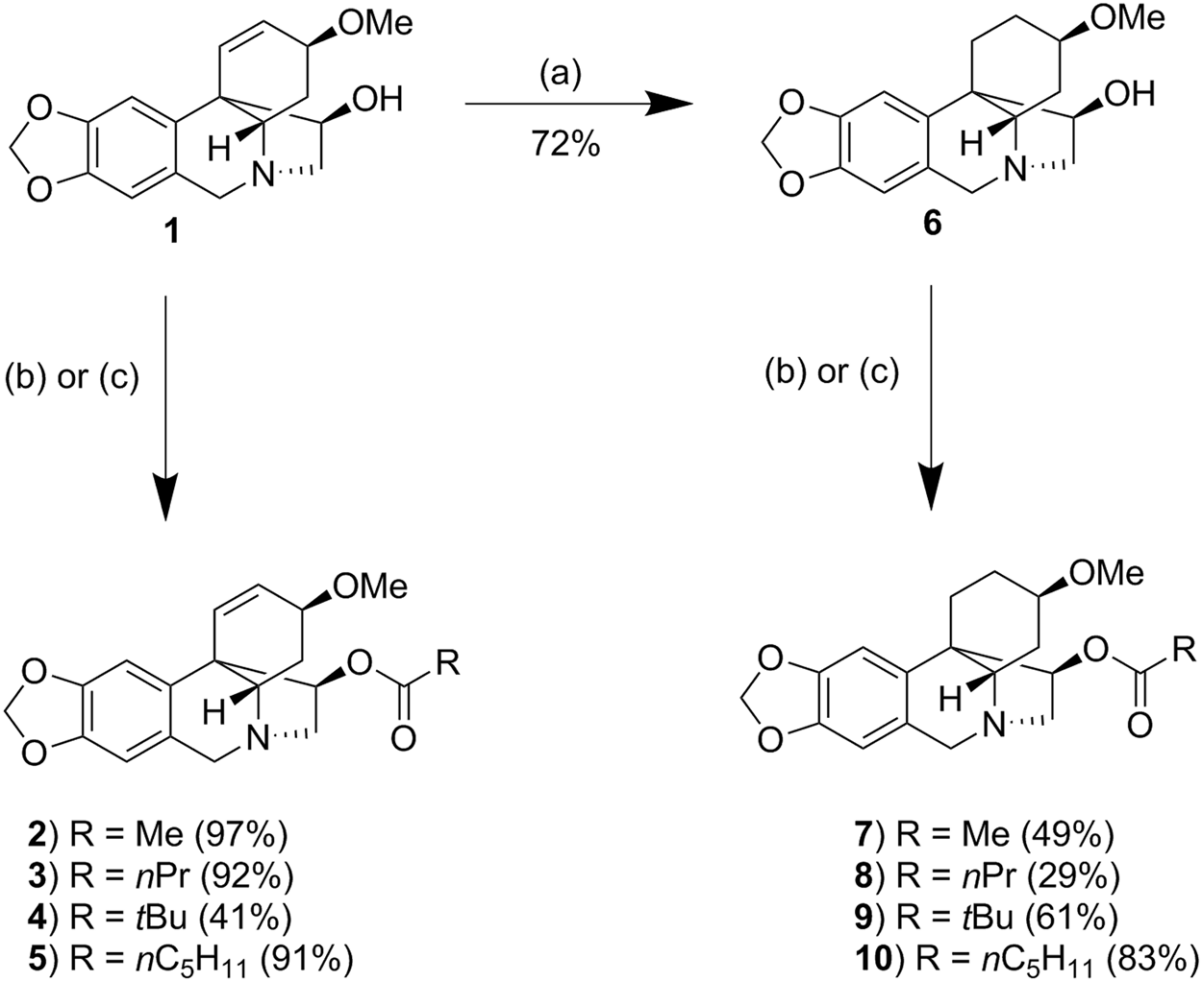
Production of derivatives from haemanthamine. (**a**) Under a nitrogen atmosphere, palladium on charcoal was added to a solution of haemanthamine **1** dissolved in dry tetrahydrofuran (THF). After evacuation, the mixture was stirred at room temperature under a hydrogen atmosphere for 16 hrs following which the reaction was filtered through a pad of Celite© which was washed with excess THF and the filtrate evaporated under reduced pressure. The residue was purified by flash column chromatography (to give dihydrohaemanthamine **6**). (**b**) Pyridine and DMAP were added to a solution of haemanthamine 1 or dihydrohaemanthamine  **6** dissolved in dichloromethane. The mixture was cooled (0 °C) and acetic anhydride or the requried acid chloride was added slowly. The resultant mixture was stirred until complete consumption of starting material. The reaction was washed with NaOH solution and brine, dried and evaporated under reduced pressure. The crude product was purified using flash column chromatography. (**c**) Triethylamine and DMAP were added to a solution of haemanthamine **1** in dichloromethane. The mixture was cooled (0 °C) and the required acid chloride was added slowly and allowed to stir until complete consumption of the starting material. The sample was then washed subsequently with NaOH solution and brine then dried and evaporated under reduced pressure. The product was then purified using flash column chromatography.

Haemanthamine **1** was extracted from a fermented bulk of daffodil plants (*Narcissus Carlton*), from which galanthamine had been previously removed, and purified by recrystallization from acetone. The purified haemanthamine **1** was then used to produce a series of analogous compounds as shown in Fig. 1. The first derivatives obtained were the four simple esters (**2**–**5**) of acetate **2**, butyrate **3**, pivalate **4** and hexanoate **5**. The second series of derivatives were prepared from dihydrohaemanthamine **6**, which was obtained by the hydrogenation of haemanthamine **1**. Esterification of **6** was achieved in a similar manner as before and gave esters of acetate **7**, butyrate **8**, pivalate **9** and hexanoate **10**. Full synthesis and purification details and yields for each step together with structures are shown in Supplementary Material and in Supplementary Fig. S1

### Antiprotozoal activity

Bacterial degradation by protozoa increased linearly (R^2^ > 0.99) over 5 h in the control incubations. For each compound, the rate of bacterial degradation (% h^−1^), a proxy of protozoal activity, at 0.125, 0.25, 0.5 and 1 g/L as compared with the control is shown in Supplementary Table S1 The inhibition of protozoa activity (Table 1) was significantly different between compounds and doses (P < 0.001). When added at 1 g/L, all compounds inhibited protozoa activity by 64–84%. Greater differences in the antiprotozoal effect between compounds were observed at lower concentrations, with dihydrohaemanthamine derivatives, and particularly derivative **10**, having a stronger effect.

**Table 1:** Inhibition of protozoa activity (% in respect to the control, no addition) by dihydrohaemanthamine and derivatives of haemanthamine and dihydrohaemanthamine, added at 0.125, 0.25, 0.5 or 1 g/L.

	Dose (g/L)			
	0.125	0.25	0.5	1
**Haemanthamine derivatives**				
2	13.9^a^	24.9^a^	38.3^b^	63.5^c^
3	0.2^a^	12.2^a^	72.1^b^	78.8^b^
4	10.7^a^	47.9^b^	79.7^c^	78.8^c^
5	7.7^a^	11.1^a^	55.8^b^	68.5^b^
**Dihydrohaemanthamine derivatives**				
6	17.4^a^	26.1^a^	42.5^b^	74.0^c^
7	3.8^a^	6.1^a^	32.9^b^	66.2^c^
8	4.8^a^	33.3^b^	81.8^c^	81.8^c^
9	17.9^a^	69.1^b^	89.3^c^	83.9^c^
10	41.7^a^	79.0^b^	78.8^bc^	83.5^c^
	SED		P	
Treatment	3.258		<0.001	
Dose	2.171		<0.001	
Treatment x Dose	6.516		<0.001	

SED: Standard error of the difference. ^a–c^Means with different superscript differ significantly by dose within treatment.

### Effect on fermentation parameters

The synthetized compounds, at 0.5 and 1 g/L, were tested further over 24 h in *in vitro* incubations (Tables 2 and 3). Overall, fermentation pattern was significantly different between compounds and doses (P < 0.001). Only total gas produced after 24 h was unaffected by the treatments, although all derivatives had an impact on methane emissions (P < 0.001).

**Table 2:** Effect of dihydrohaemanthamine and derivatives of haemanthamine and dihydrohaemanthamine (added at 0.5 and 1 g/L) on total (mM) and individual (% of total VFA) after 24 h of incubation.

	Total VFA (mM)			Acetate (%)			Propionate (%)			Butyrate (%)			BCVFA (%)		
	Dose (g/L)			Dose (g/L)			Dose (g/L)			Dose (g/L)			Dose (g/L)		
	0	0.5	1	0	0.5	1	0	0.5	1	0	0.5	1	0	0.5	1
**Haemanthamine derivatives**															
2	62.9^a^	50.7^b^	46.7^b^	58.8^a^	48.0^b^	47.4^b^	16.4^a^	24.6^b^	23.8^b^	18.5^a^	21.3^a^	22.3^a^	2.70^a^	2.56^a^	2.56^a^
3	62.9^a^	52.1^ab^	49.0^b^	58.8^a^	47.3^b^	42.9^b^	16.4^a^	23.3^b^	25.0^b^	18.5^a^	22.8^ab^	25.8^b^	2.70^a^	2.68^a^	2.55^a^
4	62.9^a^	55.6^a^	52.5^a^	58.8^a^	48.7^b^	46.0^b^	16.4^a^	23.2^b^	23.9^b^	18.5^a^	21.9^ab^	23.6^b^	2.70^a^	2.49^a^	2.41^a^
5	62.9^a^	51.7^a^	53.3^a^	58.8^a^	48.3^b^	46.6^b^	16.4^a^	23.3^b^	23.6^b^	18.5^a^	21.2^a^	22.2^a^	2.70^a^	2.79^a^	2.52^a^
**Dihydrohaemanthamine derivatives**															
6	62.9^a^	57.7^a^	52.6^a^	58.8^a^	56.4^a^	55.5^a^	16.4^a^	19.8^a^	20.2^a^	18.5^a^	17.8^a^	18.3^a^	2.70^a^	2.46^a^	2.48^a^
7	62.9^a^	51.1^b^	49.5^b^	58.8^a^	49.0^b^	47.5^b^	16.4^a^	25.0^b^	24.5^b^	18.5^a^	19.7^a^	21.8^a^	2.70^a^	2.64^a^	2.45^a^
8	62.9^a^	51.4^b^	46.9^b^	58.8^a^	45.5^b^	36.8^c^	16.4^a^	24.0^b^	26.9^b^	18.5^a^	24.0^b^	29.2^b^	2.70^a^	2.58^a^	2.05^b^
9	62.9^a^	51.3^b^	55.6^ab^	58.8^a^	44.6^b^	41.6^b^	16.4^a^	26.2^b^	33.9^c^	18.5^a^	22.0^a^	17.0^a^	2.70^a^	3.27^b^	4.18^c^
10	62.9^a^	55.6^a^	62.3^a^	58.8^a^	41.3^b^	41.2^b^	16.4^a^	27.2^b^	37.9^c^	18.5^a^	22.3^a^	10.5^b^	2.70^a^	2.28^ab^	2.05^b^
		SED	P		SED	P		SED	P		SED	P		SED	P
Treatment		1.630	0.001		1.110	<0.001		0.572	<0.001		0.643	<0.001		0.061	<0.001
Dose		0.943	<0.001		0.638	<0.001		0.330	<0.001		0.371	<0.001		0.035	0.004
Treatment x Dose		2.830	0.023		1.910	<0.001		0.991	<0.001		1.110	<0.001		0.106	<0.001

VFA: volatile fatty acids; BCVFA: branched chain volatile fatty acids; SED: Standard error of the difference. ^a-b^Means with different superscript differ significantly by dose within treatment.

**Table 3:** Effect of dihydrohaemanthamine and derivatives of haemanthamine and dihydrohaemanthamine, added at 0.5 and 1 g/L, on pH, ammonia (mM) and total gas and methane (mL) produced after 24 h of incubation.

	pH			Ammonia (mM)			Total gas (mL)			Methane (mL)		
	Dose (g/L)			Dose (g/L)			Dose (g/L)			Dose (g/L)		
	0	0.5	1	0	0.5	1	0	0.5	1	0	0.5	1
**Haemanthamine derivatives**												
2	6.19^a^	6.23^ab^	6.36^b^	7.12^a^	5.16^b^	6.06^ab^	25.1^a^	21.36^ab^	18.04^b^	2.99^a^	0.005^b^	0.003^b^
3	6.19^a^	6.29^ab^	6.38^b^	7.12^a^	6.51^a^	6.34^a^	25.1^a^	22.09^ab^	19.77^b^	2.99^a^	0.003^b^	0.003^b^
4	6.19^a^	6.17^a^	6.37^b^	7.12^a^	5.24^b^	6.17^ab^	25.1^a^	23.56^ab^	17.77^b^	2.99^a^	1.0^b^	0.48^b^
5	6.19^a^	6.29^a^	6.27^a^	7.12^a^	7.04^a^	6.07^a^	25.1^a^	22.10^ab^	21.59^a^	2.99^a^	0.003^b^	0.003^b^
**Dihydrohaemanthamine derivatives**												
6	6.19^a^	6.25^ab^	6.36^b^	7.12^a^	5.38^b^	5.85^ab^	25.1^a^	21.95^ab^	18.96^b^	2.99^a^	1.94^a^	1.62^a^
7	6.19^a^	6.31^ab^	6.34^b^	7.12^a^	5.72^b^	6.12^ab^	25.1^a^	21.61^ab^	19.61^b^	2.99^a^	0.003^b^	0.003^b^
8	6.19^a^	6.33^b^	6.37^b^	7.12^a^	6.65^a^	6.76^a^	25.1^a^	21.04^ab^	19.80^b^	2.99^a^	0.003^b^	0.003^b^
9	6.19^a^	6.31^ab^	6.33^b^	7.12^a^	6.94^a^	6.71^a^	25.1^a^	20.29^ab^	17.75^b^	2.99^a^	0.003^b^	0.003^b^
10	6.19^a^	6.26^a^	6.25^a^	7.12^a^	6.86^a^	6.08^a^	25.1^a^	19.35^b^	18.38^b^	2.99^a^	0.003^b^	0.003^b^
		SED	P		SED	P		SED	P		SED	P
Treatment		0.019	0.020		0.190	<0.001		0.730	0.176		0.251	<0.001
Dose		0.011	<0.001		0.110	<0.001		0.421	<0.001		0.145	<0.001
Treatment x Dose		0.033	<0.001		0.329	<0.001		1.26	0.330		0.434	0.154

SED: Standard error of the difference. ^a,b^Means with different superscript differ significantly by dose within treatment.

Whereas slight increases in pH (P < 0.001) were observed in the presence of the synthetized compounds, the concentration of total volatile fatty acids (VFA) decreased (P < 0.001) by 19 and 26% when they were added at 0.5 and 1 g/L, respectively. Dihydrohaemanthamine **6**, its derivatives (**7**–**10**) and haemathamine derivatives (**2**–**5**) caused shifts in the molar proportions of VFA towards lower acetate and higher propionate (P < 0.001), to different extents depending on the compound. The greatest effect was observed with the dihydrohaemanthamine derivatives, and particularly **8**, **9** and **10**. For most of the synthetized compounds, whilst molar proportions of butyrate increased (P < 0.001), those of branched chain volatile fatty acids (BCVFA) decreased (P < 0.001). All compounds caused decreases in ammonia concentration (P < 0.001), this effect being greater with dihydrohaemanthamine **6** and the derivatives of haemanthamine (**2**–**5**).

## Discussion

Alkaloids of the amaryllidaceae family have been reported to have a wide range of biological activities^8^. Some of these alkaloids are of particular interest because of their potential use in the treatment of protozoal diseases such as leshmaniasis, trypanosomiases and malaria^9^, whilst antibacterial and antifungal activities have also been described^10^.

Several studies aimed at developing antimalarial drugs on the relationship between the structure of novel synthesised compounds and their biological activity have been published^11–13^. Cedrón *et al*.^14^ observed that haemanthamine derivatives with a methoxy group at C-3 and the presence of a free hydroxyl group at C-11 were more effective against protozoa than other derivatives; this antiprotozoal effect was also associated with the presence of a double bond at C1-C2^14^.

The aim of this work was to study the relationship between the chemical structure of certain compounds and their effects on rumen protozoa and fermentation pattern; thus, nine compounds were synthetized from the natural alkaloid haemanthamine. The chemical modifications were carried out on the hydroxyl group at C-11 and/or on the double bond presents at C1-C2 in ring D. The hydrogenation of the double bond at C1-C2 of haemanthamine resulted in the derivative dihydrohaemanthamine **6**. Haemanthamine (**2**–**5**) or dihydrohaemanthamine (**7**–**10**) derivatives were obtained by esterification of the hydroxyl group at C-11 with acetate (**2** and **7**), butyrate (**3** and **8**), pivalate (**4** and **9**) or hexanoate (**5** and **10**). ^1^H NMR and ^13^C NMR spectra of each compound are shown in Supplementary Information.

All the compounds tested showed antiprotozoal effect that differed between treatments and doses, with dihydrohaemanthamine derivatives being more effective in inhibiting protozoal activity. Since the simple esters made the derivatives more lipophilic than the non-esterified molecule, it may have allowed them to cross the cell membrane of protozoa increasing then their antiprotozoal activity. The esterification of the hydroxyl group of dihydrohaemanthamine with hexanoate (derivative **10**) increased the antiprotozoal effect dramatically. However, the same modification of the haemanthamine molecule (derivative **5**) reduced the antiprotozoal effect. This decrease in activity, particularly at the lowest doses tested, was also observed with haemanthamine derivatives obtained by esterification with butyrate and pivalate (**3** and **4**) as compared with those of dihydrohaemanthamine with the same modifications in the structure (**8** and **9**). However, when the substituent was acetate, dihydrohaemanthamine derivative **7** was less effective inhibiting protozoa than the corresponding haemanthamine derivative **2** or the dihydrohaemanthemine molecule. Our results indicate that the double bond present at C1-C2 in ring D may only play a role in the antiprotozoal activity when combined with certain chemical modifications in the structure.

Although the concentration of total VFA was reduced by the synthetized compounds, as compared with the control (no derivative added), a shift in the molar proportion of VFA towards propionate and, to a lesser extend butyrate, at expenses of acetate was observed across treatments. While the increase in propionate was substantial with **9** and **10** (33.9 and 37.9%, respectively, vs 16.4%), increases in butyrate were not observed in incubations with these compounds.

Dihydrohaemanthamine derivatives had the greatest effect on the molar proportions of VFA, which is in line with the effects observed on protozoa activity, followed by haemanthamine derivatives and dihydrohaemanthamine. Similar effects on molar proportions of VFA were observed when acetate was esterified to haemanthamine or dihydrohaemanthamine, contrary to what was observed when studying protozoal activity.

Interestingly, dihydrohaemanthamine and derivatives of haemanthamine were more effective in reducing ammonia concentration (by 18–24% and 9–27%, respectively) than dihydrohaemanthamine derivatives, being **8** and **9** the less effective compounds (2.5–6% reduction). The reduction in ammonia in the presence of the derivatives could be explained by the observed effect on protozoa, involved in the turnover of bacterial protein in the rumen^15^, and possibly an inhibitory effect on high ammonia-producing bacteria. The esterification of the dihydrohaemanthamine molecule, however, decreased the ability of the compounds to reduce ammonia, as compared with that observed for dihydrohaemantamine.

All derivatives seemed to have a remarkable effect on methane emissions; whereas dihydrohaemanthemine **6** and compound **4** decreased methane by 35–45% and 66–84%, respectively, the rest of derivatives caused almost the complete inhibition of methane production, even at the lowest concentration tested. This may suggest a direct effect of the derivatives on the population of methanogenic archaea.

**Figure 2:**
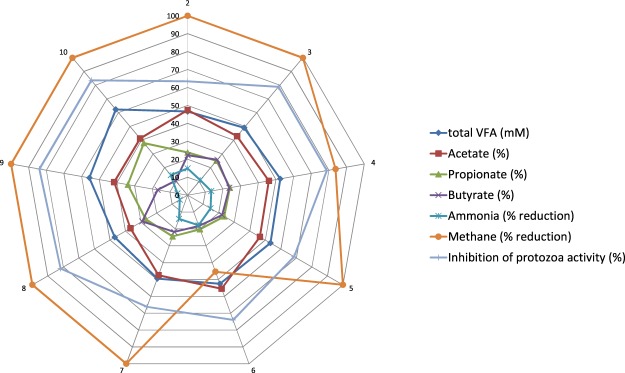
Effect of dihydrohaemanthamine **6** and derivatives of haemanthamine (**2–5**) and dihydrohaemanthamine (**7–10**), added at 1 g/L, on fermentation pattern after 24 h of incubation. Each axis represents one compound tested. Each point in the plot represents a value for total VFA (mM), molar proportions of acetate, propionate and butyrate, ammonia or methane reduction (percentage in respect to the control, no addition) and inhibition of protozoa (percentage in respect to the control, no addition).

The different effects of the compounds added at 1 g/L on fermentation parameters are illustrated in Fig. 2 in which molar proportions of VFA, the percentage of reduction of ammonia and methane, and the percentage of inhibition of protozoa activity are plotted in a figure with 9 axes, one for each compound. The esterification of dihydrohaemanthamine with hexanoate, acetate, butyrate or pivalate led to a greater effect on VFA, although it did not seem to have the same impact on ammonia concentration, as compared with the effects observed with the rest of derivatives. The esterification of either haemanthamine or dihydrohaemanthamine did not seem to enhance the inhibitory effect on ammonia, as a greater reduction was observed with dihydrohaemanthamine. All the esterified compounds, however, were more effective in decreasing acetate and increasing propionate molar proportions than the dihydrohaemanthamine molecule. All derivatives, when added at 1 g/L, showed a great antiprotozoal effect and inhibited, almost completely, methane production.

It appears that whilst certain modifications in the chemical structure may influence some aspect of rumen fermentation, they may not necessary have an effect on others. Since the stability of the compounds overtime was not evaluated, it cannot be ruled out that the observed effects on fermentation could be also due to the potential breakdown products obtained from these derivatives. Studies to characterize the changes in rumen microbial communities associated with the effects of these derivatives on rumen fermentation are needed to understand which structural features may play a role in improved feed efficiency. The evaluation of the effects of other substituents and/or more than one variant in the structure as well as the synergistic effects is also suggested. However, it is clear that relative small variations in the structure of a compound can have a significant influence on their biological activity in terms of the ability to manipulate gut fermentation. Thus, future studies on the effect of plant extracts on fermentation in the gut of farm and companion animals need to combine detailed chemical characterisation of the extract used together with examination of the biological effects in the gut.

## Methods

### Synthesis of haemanthamine derivatives

Haemanthamine (extracted from Narcissus Carlton) was provided by Agroceutical Ltd. Synthesis pathways used are summarised in Fig. 1 and described in detail in the Supplementary Information.

The purity of the synthesised compounds was established by quantitative nuclear magnetic resonance (qNMR) spectroscopy using a Bruker Ultrashielded 400 spectrometer (Bruker Corporation, Coventry, UK) confirming purities of >98% for all derivatives.

### Measurement of protozoal activity

The effect of dihydrohaemanthamine and derivatives of haemanthamine and dihydrohaemanthamine on protozoal activity was measured *in vitro* as the breakdown of [^14^C]-labelled bacteria by rumen protozoa as described by Wallace and McPherson^16^. Isotope-labelled bacteria were obtained by growing *Streptococcus bovis* ES1 in Wallace and McPherson media^16^ containing [^14^C] leucine (1.89 µCi/7.5 mL tube) as the sole nitrogen source, for 24 h. Cultures were centrifuged (3,000 g, 15 min), supernatant discarded and pellets re-suspended in 7 mL of simplex type salt solution^17^ (STS) containing ^12^C-leucine (5 mM). This process was repeated three times to prevent re-incorporation of released [^14^C] leucine by bacteria.

Rumen digesta was obtained from four rumen-cannulated Holstein-Frisian cows (four replicates) fed at maintenance level (composed of perennial ryegrass hay and concentrate at 67:33 on a DM basis). Animal procedures were carried out in accordance with the Animal Scientific Procedures Act 1986 and protocols were approved by the Aberystwyth University Ethical Committee. Rumen digesta was obtained before the morning feeding and strained through two layers of muslin and diluted with STS (1:1) containing ^12^C-leucine (5 mM). Diluted rumen fluid (7.5 mL) was then incubated with labelled bacteria prepared as described above (0.5 mL) in tubes containing no additive (control) or 0.125, 0.25, 0.5 or 1 g/L of the compounds. Incubations were carried out at 39 °C under a stream of CO_2_ and tubes were sampled at time 0 and at 1 h intervals up to 5 h using a syringe with a 19 gauge needle. Samples (0.5 mL) were acidified (by adding 0.125 mL of 25% trichloroacetic acid (wt/vol) and centrifuged (13,000 *g*, 5 min). Supernatant (0.200 mL), was diluted with 2 mL of OptiPhase HiSafe 2 scintillation fluid (Perkin Elmer, Seer Green, UK) to determine the radioactivity released by liquid-scintillation spectrometry (Hidex 300 SL, Lablogic Systems Ltd, Broomhill, UK). Bacterial breakdown at each incubation time was expressed as the percentage of the acid-soluble radioactivity released relative to the total radioactivity present in the initial labelled bacteria^16^.

A simple linear regression was conducted to model the relationship between the percentage of radioactivity released (relative to the ^14^C-bacterial inoculum) and the time (from 0 h to 5 h), as well as its correlation coefficient. The slope of this trend-line indicated the bacterial degradation rate (as % h^−1^) and ultimately acts as a proxy of protozoal activity.

### Determination of rumen fermentation pattern

To measure the short term effect of dihydrohaemanthamine and derivatives of haemanthamine and dihydrohaemanthamine on fermentation parameters, 24 h *in vitro* incubations were carried out. The experimental design consisted of a control (no additive) and the compounds added at 0.5 or 1 g/L. The experiment was conducted in quadruplicate, using rumen fluid from the same four cannulated cows. Rumen contents were sampled before the morning feeding, filtered through a double layer of muslin and diluted 1:2 in artificial saliva solution^18^. Aliquots (10 mL) of the diluted strained rumen fluid were added anaerobically to 40 mL Wheaton bottles containing 0.1 g of diet composed of ryegrass hay and barley (40:60), previously ground to pass through a 1-mm^2^ mesh screen. Bottles were sealed and incubated at 39 °C receiving a gentle mix before sampling.

After 24 h of the incubation, gas was measured using a pressure transducer. After pressure was released, a gas sample (0. 5 mL) was collected from the headspace and immediately injected in a chromatograph (ATI Unicam 610 Series, Unicam Ltd., Cambridge, UK), fitted with a 40 cm Porapak N metal packed column (Agilent, Cheshire, UK) and flame ionization detector, to determine methane concentration. Then, bottles were opened, pH measured and a sample was collected and divided in two subsamples: one of the subsamples (4 mL) was diluted with 1 mL of deproteinising solution (200 mL/L orthophosphoric acid containing 20 mmol/L of 2-ethylbutyric acid as an internal standard) for the determination of VFA using gas chromatography, as described by Stewart and Duncan^19^. Another subsample (1 mL) was diluted with 0.250 mL of 25% trichloroacetic acid (wt/vol) for analysis of ammonia using a colorimetric method^20^.

### Statistical analyses

Trend line slopes were analysed statistically by randomized block ANOVA, with individual cows as a blocking term. Inhibition of protozoa activity (% with respect to the control) and fermentation parameters were analysed using ANOVA with treatment, dose and their interaction as fixed effects and cow as blocking term. When significant effects were detected across the different doses, means were compared by Fisher’s unprotected LSD test.

## Supplementary information

Supplementary information

## Data Availability

All data generated or analysed during this study are included in the published article (and its Supplementary Information files).
