# Supplementary material for: Slight changes in the chemical structure of haemanthamine greatly influence the effect of the derivatives on rumen fermentation in vitro
Source: Sci Rep. 2019 Feb 21;9:2440. doi: 10.1038/s41598-019-38977-x (PMC6385227; doi:10.1038/s41598-019-38977-x)
Supplement: Supplementary file 1 — Supplementary information [file 41598_2019_38977_MOESM1_ESM.pdf]

Supplementary information

Slight changes in the chemical structure of haemanthamine greatly influence the effect of the derivatives on rumen fermentation in vitro

Eva Ramos-Morales[1*], Jamie Tibble-Howlings[2], Laura Lyons[3], Patrick J. Murphy[2], Radek Braganca[2], Magnus O. Ogbu[2] and Charles James Newbold[1]

Synthesis of compounds 2-10

Compound 2. (3S,4aS,11bS,12R)-3-methoxy-4,4a-dihydro-3H,6H-11b,5-ethano[1,3]dioxolo[4,5-j]phenanthridin-12-yl acetate

Pyridine (1.34 mL, 16.6 mmol) and DMAP (10 mg) were added to a solution of haemanthamine **1** (1.00 g, 3.32 mmol) dissolved in dichloromethane (20 mL). The mixture was cooled (0 °C) and acetic anhydride (0.94 mL, 9.96 mmol) was added slowly. The resultant mixture was stirred until complete consumption of starting material. The reaction was washed with NaOH solution (1 M, 3 x 30 mL) and brine (3 x 30 mL), dried ($MgSO_4$) and evaporated under reduced pressure. The crude product was purified using silica gel flash chromatography (1-5% MeOH in chloroform, gradient elution) to give **2** (1.10 g, 3.20 mmol, 97% yield) as a gum. Rf = 0.48 (10% MeOH in DCM); ^{1}H NMR (400 MHz, $CDCl_3$): δ 6.89 (1H, s, CH), 6.46 (1H, s, CH), 6.34 (1H, d, *J* 10.1 Hz, CH), 6.16 (1H, dd, *J* 4.6, 10.1 Hz, CH), 5.89 (2H, s, CH_2), 4.96 (1H, dd, *J* 3.4, 7.0 Hz, CH), 4.35 (1H, d, *J* 16.8 Hz, CH), 3.83-3.86 (1H, m, CH), 3.73 (1H, d, *J* 16.8), 3.40 (1H, dd, *J* 7.2, 14.5, CH), 3.53 (3H, s, CH_3), 3.13 (1H, dd, *J* 3.6, 14.5 Hz, CH), 2.05 (1H, dd, *J* 4.5, 13.6, CH), 1.97 (3H, s, CH_3), 1.92 (1H, dd, *J* 5.2, 13.6 Hz, CH); ^{13}C NMR (125MHz, $CDCl_3$): δ 170.2, 146.8, 146.6, 134.4, 129.7, 127.8, 126.5, 106.7, 104.1, 101.0, 80.5, 77.2, 72.6, 63.0, 61.3, 60.7, 56.7, 49.3, 28.5, 21.4 ppm; ν_{max} 2932, 2821, 1737, 1483, 1240, 1035, 754 cm^{-1}; MS(CI) 344.2 (100%, [M+H$^+$]) 687.3 (10%, [2M+H$^+$]); HRMS(ES) found 344.1490, $C_{19}H_{22}O_5N$ ([M+H$^+$]) requires 344.1492.

Compound 3. (3S,4aS,11bS,12R)-3-methoxy-4,4a-dihydro-3H,6H-11b,5-ethano[1,3]dioxolo[4,5-j]phenanthridin-12-yl butyrate

Pyridine (1.34 mL, 16.6 mmol) and DMAP (10 mg) were added to a solution of haemanthamine **1** (1.00 g, 3.32 mmol) dissolved in dichloromethane (20 mL). The mixture was cooled (0 °C) and *n*-butanoyl chloride (1.03 mL, 9.96 mmol) was added slowly. The resultant mixture was stirred until complete consumption of starting material. The reaction was washed with NaOH solution (1 M, 3 x 30 mL) and brine (3 x 30 mL), dried ($MgSO_4$) and evaporated under reduced pressure. The crude product was purified using silica gel flash chromatography (1-5% MeOH in chloroform, gradient elution) to give **3** (1.125 g, 3.03 mmol, 92% yield) as a gum. Rf = 0.53 (10% MeOH in DCM); ^{1}H NMR (400 MHz, $CDCl_3$): δ 6.88 (1H, s, CH), 6.44 (1H, s, CH), 6.33 (1H, d,

J 10.1 Hz, CH), 6.12 (1H, dd, *J* 5.0, 10.0 Hz, CH), 5.87 (2H, s, CH_2), 4.96 (1H, dd, *J* 3.5, 7.1 Hz, CH), 4.33 (1H, d, *J* 16.9, CH), 3.83-3.79 (1H, m, CH), 3.69 (1H, d, *J* 16.9 Hz, CH), 3.41-3.53 (1H, m, CH), 3.34 (3H, s, CH_3), 3.28 (1H, dd, *J* 3.5, 14.3 Hz, CH), 2.18 (2H, td, *J* 2.7, 7.4 Hz, CH_2), 2.02 (1H, dd, *J* 4.8, 13.6 Hz, CH), 1.92 (1H, td, *J* 4.2, 13.4 Hz, CH), 1.57 (2H, hextet, *J* 7.39 Hz, CH_2), 0.91 (3H, t, *J* 7.4 Hz, CH_3); ^{13}C NMR (125 MHz, $CDCl_3$): δ 172.7, 146.7, 146.5, 134.5, 129.6, 127.9, 126.7, 106.7, 104.0, 101.0, 80.2, 72.7, 62.9, 61.3, 60.8, 56.6, 49.3, 36.5, 28.5, 18.4, 13.8 ppm; ν_{max} 3020, 2935, 2401, 1730, 1483, 1216, 755 cm^{-1}; MS(CI) 372.2 (100%, $[M+H^+]$) 743.4 (15%, $[2M+H^+]$); HRMS(ES) found 372.1801, $C_{21}H_{26}O_5N$ ($[M+H^+]$) requires 372.1805

Compound 4. (3S,4aS,11bS,12R)-3-methoxy-4,4a-dihydro-3H,6H-11b,5-ethano[1,3]dioxolo[4,5-j]phenanthridin-12-yl pivalate

Triethylamine (1.39 mL, 9.96 mmol) and DMAP (10 mg) were added to a solution of haemanthamine **1** (1.0 g, 3.32 mmol) in dichloromethane (20 mL). The mixture was cooled (0 °C) and pivaloyl chloride (1.22 mL, 9.96 mmol) was added slowly and allowed to stir until complete consumption of the starting material. The sample was then washed subsequently with 1 M NaOH solution (3 x 30 mL) and brine (3 x 30 mL) then dried ($MgSO_4$) and evaporated under reduced pressure. The product was then purified using flash column chromatography (1-10% MeOH in chloroform, gradient elution) to give **4** (520 mg, 1.35 mmol, 41% yield) as a gum. Rf = 0.61 (10% MeOH in DCM); ^1H NMR (400 MHz, $CDCl_3$): δ 6.91 (1H, s, CH), 6.47 (1H, s, CH), 6.35 (1H, d, *J* 10.1 Hz, CH), 6.15 (1H, dd, *J* 5.0, 10.0 Hz, CH), 5.89 (2H, s, CH_2), 4.92 (1H, dd, *J* 3.3, 7.1 Hz, CH), 4.36 (1H, d, *J* 16.9 Hz, CH), 3.80-3.86 (1H, m, CH), 3.72 (1H, d, *J* 6.9 Hz, CH), 3.38-3.43 (1H, m, CH), 3.36 (3H, s, CH_3), 3.26 (1H, dd, *J* 3.3, 14.3 Hz, CH), 2.05 (1H, dd, *J* 4.5, 13.7 Hz, CH), 1.94 (1H, td, *J* 4.2, 13.5 Hz, CH), 1.14 (9H, s, 3 x CH_3); ^{13}C NMR (125 MHz, $CDCl_3$): δ 177.5, 146.7, 146.5, 134.4, 129.5, 127.9, 126.6, 106.7, 104.0, 101.0, 79.9, 72.8, 62.9, 61.3, 60.9, 56.6, 53.5, 49.3, 28.4, 27.2 ppm; ν_{max} 2930, 1725, 1483, 1156 cm^{-1}; MS(CI) 386.2 (100%, $[M+H^+]$) ; HRMS(ES) found 386.1954, $C_{22}H_{28}O_5N$ ($[M+H^+]$) requires 386.1962

Compound 5. (3S,4aS,11bS,12R)-3-methoxy-4,4a-dihydro-3H,6H-11b,5-ethano[1,3]dioxolo[4,5-j]phenanthridin-12-yl hexanoate

Triethylamine (1.39 mL, 9.96 mmol) and DMAP (10 mg) were added to a solution of haemanthamine **1** (1.0 g, 3.32 mmol) in dichloromethane (20 mL). The mixture was cooled (0 °C) and n-hexanoyl chloride (1.39 mL, 9.96 mmol) was added slowly and allowed to stir until complete consumption of the starting material. The sample was then washed subsequently with 1 M NaOH solution (3 x 30 mL) and brine (3 x 30 mL) then dried ($MgSO_4$) and evaporated under reduced pressure. The product was then purified using flash column chromatography (1-10% MeOH in chloroform, gradient elution) to give **5** (1.146 g, 3.03 mmol, 91% yield) as a gum. Rf = 0.73 (10% MeOH in DCM); ^1H NMR (400 MHz, $CDCl_3$): δ 6.89 (1H, s, CH), 6.45 (1H, s, CH), 6.33 (1H, d, *J* 10.1 Hz,CH), 6.13 (1H, dd, *J* 5.0, 10.0 Hz, CH), 5.87 (2H, s, CH_2), 4.96 (1H, dd, *J* 3.5, 7.0

Hz, CH), 4.34 (1H, d, J 16.8Hz, CH), 3.79-3.84 (1H, m, CH), 3.70 (1H, d, J 16.8 Hz, CH), 3.39 (1H, dd, J 7.1, 15.4 Hz, CH), 3.34 (3H, s, CH_3), 3.29 (1H, dd, J 3.5, 14.4, CH), 2.19 (2H, td, J 2.7, 7.4 Hz, CH_2), 2.03 (1H, dd, J 4.3, 13.6 Hz, CH), 2.03 (1H, dd, J 4.3, 13.6 Hz, CH), 1.93 (1H, td, J 4.2, 12.4 Hz, CH), 1.54 (2H, p, J 7.44 Hz, CH_2), 1.22-1.33 (4H, m, 2 x CH_2), 0.88 (3H, t, J 6.8Hz, CH_3); ^{13}C NMR (125 MHz, $CDCl_3$): δ 172.9, 146.8, 146.6, 134.5, 129.6, 128.0, 126.7, 106.7, 104.1, 101.0, 80.3, 72.7, 62.9, 61.4, 60.8, 56.7, 49.3, 34.6, 31.4, 28.5, 24.7, 22.4, 14.0 ppm; ν_{max} 2957, 2871, 1736, 1484, 1240, 1091, 755 cm^{-1}; MS(CI) 400.2 (55%, [M+H^+]); HRMS(ES) found 400.2115, $C_{23}H_{30}O_5N$ ([M+H^+]) requires 400.2118

Compound 6. (3R,4aS,11bS,12R)-3-methoxy-2,3,4,4a-tetrahydro-1H,6H-11b,5-ethano[1,3]dioxolo[4,5-j]phenanthridin-12-ol

Under a nitrogen atmosphere, palladium on charcoal (10%, 0.1 g) was added to a solution of haemanthamine **1** (5.00 g, 16.6 mmol) dissolved in dry THF (100 mL). After evacuation, the mixture was stirred at rt under a hydrogen atmosphere for 16 hrs following which the reaction was filtered through a celite pad which was washed with excess THF and the filtrate evaporated under reduced pressure. The residue was purified by flash column chromatography (1-5% MeOH in chloroform, graduated elution) to give dihydrohaemanthamine **6** (3.62 g, 11.9 mmol, 72% yield) as a white crystalline solid. Rf = 0.17 ((10% MeOH in DCM); ^1H NMR (400 MHz, $CDCl_3$): δ 6.69 (1H, s, CH), 6.39 (1H, s, CH), 5.86 (2H, s, CH_2), 4.26 (1H, d, J 16.70 Hz,CH), 4.10-4.04 (1H, m, CH), 3.69 (1H, s, CH), 3.64 (1H, d, J 16.71 Hz, CH), 3.35-3.19 (7H, m), 2.34-1.75 (6H, m); ^{13}C NMR (125 MHz, $CDCl_3$): δ 146.3, 145.6, 139.2, 125.7, 105.9, 103.3, 100.5, 82.1, 75.4, 62.8, 62.4, 60.6, 55.4, 45.9, 29.9, 26.7, 22.6 ppm; ν_{max} 3028, 2924, 1509, 1228, 1045, 822, 724 cm^{-1}; MS(CI) 304.1 (100%, [M+H^+]), 607.3 (5%, [2M+H^+]); HRMS(ES) found 304.1540, $C_{17}H_{22}O_5N$ ([M+H^+]) requires 304.1543

Compound 7. (3R,4aS,11bS,12R)-3-methoxy-2,3,4,4a-tetrahydro-1H,6H-11b,5-ethano[1,3]dioxolo[4,5-j]phenanthridin-12-yl acetate

Pyridine (1.34 mL, 16.6 mmol) and DMAP (10 mg) were added to a solution of dihydrohaemanthamine **6** (1.00 g, 3.32 mmol) dissolved in dichloromethane (20 mL). The mixture was cooled (0 °C) and acetic anhydride (0.94 mL, 9.96 mmol) was added slowly. The resultant mixture was stirred until complete consumption of starting material. The reaction was washed with NaOH solution (1 M, 3 x 30 mL) and brine (3 x 30 mL), dried ($MgSO_4$) and evaporated under reduced pressure. The crude product was purified using silica gel flash chromatography (1-5% MeOH in chloroform, gradient elution) to give **7** (354 mg, 1.02 mmol, 49% yield) as a gum. Rf = 0.44 (10% MeOH in DCM); ^1H NMR (400 MHz, $CDCl_3$): δ 6.76 (1H, s, CH), 6.56 (1H, s, CH), 5.94 (2H, d, J 3.0 Hz, CH_2), 4.81 (1H, dd, J 3.6, 7.5 Hz, CH), 4.13 (1H, d, J 17.0 Hz, CH), 3.66 (1H, s, CH), 3.61 (1H, d, J 17.1 Hz, CH), 3.33 (4H, s, 2 x CH_2), 3.23-3.31 (1H, m, CH), 3.20 (2H, s, CH_2), 2.98 (2H, m, CH_2), 2.19-2.33 (1H, m, CH), 1.79-1.93 (4H, m, 2 x CH_2), 1.53 (1H, td, J 1.2, 14.4 Hz, CH); ^{13}C NMR (125 MHz, $CDCl_3$): δ 170.1, 146.7, 146.5,

134.4, 129,5, 127.7, 126.6, 106.7, 104.0, 100.9, 80.5, 72.6, 62.9, 61.3, 60.6, 56.6, 49.3, 28.4, 21.3 ppm; ν_{max} 3020, 1216, 756 cm^{-1}; MS(CI) 346.2 (100%, [$M+H^+$]), 691.3 (5% [$2M+H^+$]); HRMS(ES) found 346.1641, $C_{19}H_{24}O_5N$ ([$M+H^+$]) requires 346.1649

Compound 8. (3R,4aS,11bS,12R)-3-methoxy-2,3,4,4a-tetrahydro-1H,6H-11b,5-ethano[1,3]dioxolo[4,5-j]phenanthridin-12-yl butyrate

Pyridine (1.34 mL, 16.6 mmol) and DMAP (10 mg) were added to a solution of dihydrohaemanthamine **6** (1.00 g, 3.32 mmol) dissolved in dichloromethane (20 mL). The mixture was cooled (0 °C) and *n*-butanoyl chloride (1.03 mL, 9.96 mmol) was added slowly. The resultant mixture was stirred until complete consumption of starting material. The reaction was washed with NaOH solution (1 M, 3 x 30 mL) and brine (3 x 30 mL), dried ($MgSO_4$) and evaporated under reduced pressure. The crude product was purified using silica gel flash chromatography (1-5% MeOH in chloroform, gradient elution) to give **8** (232 mg, 0.62 mmol, 29% yield) as a gum. Rf = 0.49 (10% MeOH in DCM); ^{1}H NMR (400 MHz, $CDCl_3$): δ 6.71 (1H, s, CH), 6.40 (1H, s, CH), 5.86 (2H, s, CH_2), 5.00 (1H, dd, *J* 3.4, 6.9 Hz, CH), 4.28 (1H, d, *J* 16.9 Hz, CH), 3.70 (1H, s, CH), 3.65 (1H, d, *J* 16.8 Hz, CH), 3.37 (1H, dd, *J* 7.1, 14.3 Hz, CH), 3.27 (3H, s, CH_3), 3.12 (1H, dd, *J* 3.5, 14.5 Hz, CH), 2.27 (3H, t, *J* 7.3 Hz, CH_3), 1.80-2.10 (5H, m), 1.56-1.68 (3H, m), 1.24 (1H, t, *J* 7.1 Hz, CH), 0.94 (3H, t, *J* 7.4 Hz, CH_3); ^{13}C NMR (125 MHz, $CDCl_3$): δ 173.0, 146.7, 146.3, 138.4, 126.3, 106.1, 104.0, 100.9, 82.5, 75.3, 62.8, 61.4, 61.0, 55.8, 45.1, 36.6, 30.4, 26.8, 22.6, 18.5, 13.7; ν_{max} 3020, 2401, 1216, 757, 669 cm^{-1}; MS(CI) 374.2 (100%, [$M+H^+$]), 747.4 (10%, [$2M+H^+$]); HRMS(ES) found 374.1958, $C_{21}H_{28}O_5N$ ([$M+H^+$]) requires 374.1962.

Compound 9. (3R,4aS,11bS,12R)-3-methoxy-2,3,4,4a-tetrahydro-1H,6H-11b,5-ethano[1,3]dioxolo[4,5-j]phenanthridin-12-yl pivalate

Pyridine (1.34 mL, 16.6 mmol) and DMAP (10 mg) were added to a solution of dihydrohaemanthamine **6** (1.00 g, 3.32 mmol) dissolved in dichloromethane (20 mL). The mixture was cooled (0 °C) and pivolyl chloride (1.22 mL, 9.96 mmol) was added slowly. The resultant mixture was stirred until complete consumption of starting material. The reaction was washed with NaOH solution (1 M, 3 x 30 mL) and brine (3 x 30 mL), dried ($MgSO_4$) and evaporated under reduced pressure. The crude product was purified using silica gel flash chromatography (1-5% MeOH in chloroform, gradient elution) to give **9** (554 mg, 1.31 mmol, 61% yield) as a gum. Rf = 0.51 (10% MeOH in DCM); ^{1}H NMR (400 MHz, $CDCl_3$): δ 6.73 (1H, s, CH), 6.41 (1H, s, CH), 5.87 (2H, s, CH_2), 4.88 (1H, dd, *J* 3.2, 6.7 Hz, CH), 4.31 (1H, d, *J* 16.8 Hz, CH), 3.73 (1H, s, CH), 3.67 (1H, d, *J* 15.9 Hz, CH), 3.41 (1H, dd, *J* 7.0, 14.5 Hz, CH), 3.29 (3H, s, CH_3), 3.05 (1H, dd, *J* 3.4, 14.5, CH), 2.32-2.29 (1H, m, CH), 1.98-2.13 (4H, m, 2 x CH_2), 1.57 (1H, t, *J* 13.3 Hz, CH), 1.19 (9H, s, 3 x CH_3); ^{13}C NMR (125 MHz, $CDCl_3$): δ 178.1, 146.8, 146.4, 138.6, 126.3, 106.2, 104.1, 100.9, 83.0, 75.3, 62.9, 62.0, 61.0, 55.9, 45.1, 38.7, 30.4, 27.4, 26.8, 22.5 ppm; ν_{max} 3020, 1216, 760 cm^{-1}; MS(CI) 388.2 (100%, [$M+H^+$]),

775.4 (15%, [2M+H$^+$]); HRMS(ES) found 388.2113, $C_{22}H_{30}O_5N$ ([M+H$^+$]) requires 388.2118.

Compound 10. (3R,4aS,11bS,12R)-3-methoxy-2,3,4,4a-tetrahydro-1H,6H-11b,5-ethano[1,3]dioxolo[4,5-j]phenanthridin-12-yl hexanoate

Pyridine (1.34 mL, 16.6 mmol) and DMAP (10 mg) were added to a solution of dihydrohaemanthamine **6** (1.00 g, 3.32 mmol) dissolved in dichloromethane (20 mL). The mixture was cooled (0 °C) and n-hexanoyl chloride (1.39 mL, 9.96 mmol) was added slowly. The resultant mixture was stirred until complete consumption of starting material. The reaction was washed with NaOH solution (1 M, 3 x 30 mL) and brine (3 x 30 mL), dried ($MgSO_4$) and evaporated under reduced pressure. The crude product was purified using silica gel flash chromatography (1-5% MeOH in chloroform, gradient elution) to give 10 (711 mg, 1.77 mmol, 83% yield) as a gum. Rf = 0.55 (10% MeOH in DCM); ^{1}H NMR (400 MHz, $CDCl_3$): δ 6.72 (1H, s, CH), 6.41 (1H, s, CH), 5.87 (2H, s, CH_2), 5.02 (1H, dd, *J* 3.4, 7.0 Hz, CH), 4.32 (1H, d, *J* 16.8, CH), 3.69 (2H, d, *J* 16.6 Hz, CH_2), 3.30-3.40 (2H, m, CH_2), 3.27 (3H, s, CH_3), 3.20 (1H, dd, *J* 3.4, 8.5 Hz, CH), 2.15-2.30 (4H, m), 1.81-2.15 (4H, m), 1.57-1.65 (4H, m), 1.24-1.36 (6H, m), 0.88 (3H, t, *J* 6.9 Hz, CH_3); ^{13}C NMR (125 MHz, $CDCl_3$): δ 173.2, 146.7, 146.3, 138.5, 126.5, 106.1, 104.0, 100.9, 82.6, 75.4, 62.9, 61.6, 61.1, 55.8, 45.2, 34.7, 31.4, 30.5, 26.8, 24.8, 22.6, 22.4, 14.0 ppm; ν_{max} 3020, 2933, 1729, 1482, 1216, 757, 699 cm^{-1}; MS(CI) 402.2 (100%, [M+H$^+$]); HRMS(ES) found 402.2266, $C_{23}H_{32}O_5N$ ([M+H$^+$]) requires 402.2275

Supplementary Table S1. Effect of dihydrohaemanthamine **6** and derivatives of haemanthamine **1** and dihydrohaemanthamine **6**, added at 0.125, 0.250, 0.5 or 1 g/L, on rumen protozoa activity assessed in vitro as the amount of 14C-labelled bacteria broken down by rumen protozoa (% of the initial radioactivity released per hour)

	Dose (g/L)						
	0	0.125	0.25	0.5	1	SED	P
Haemanthamine derivatives							
2	1.44^{d}	1.22^{cd}	1.09^{bc}	0.871^{b}	0.495^{a}	0.123	<.001
3	1.44^{b}	1.58^{b}	1.32^{b}	0.383^{a}	0.270^{a}	0.154	<.001
4	1.44^{c}	1.34^{c}	0.735^{b}	0.277^{a}	0.280^{a}	0.128	<.001
5	1.44^{b}	1.42^{b}	1.25^{b}	0.653^{a}	0.426^{a}	0.130	<.001
Dihydrohaemanthamine derivatives							
6	1.44^{d}	1.17^{c}	1.06^{c}	0.810^{b}	0.363^{a}	0.111	<.001
7	1.44c	1.51c	1.39c	0.958b	0.483a	0.123	<.001
8	1.44^{c}	1.38^{c}	0.942^{b}	0.249^{a}	0.249^{a}	0.117	<.001
9	1.44^{b}	1.19^{b}	0.438^{a}	0.137^{a}	0.201^{a}	0.161	<.001
10	1.44^{c}	0.844^{b}	0.299^{a}	0.279^{a}	0.219^{a}	0.151	<.001

[a-d] Means with different superscript differ (*n*=4)

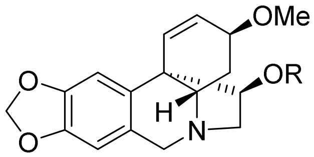

No	R	Name
**1**	H	(3S,4aS,11bS,12R)-3-methoxy-4,4a-dihydro-3H,6H-11b,5-ethano[1,3]dioxolo[4,5-j]phenanthridin-12-ol
**2**	Ac	(3S,4aS,11bS,12R)-3-methoxy-4,4a-dihydro-3H,6H-11b,5-ethano[1,3]dioxolo[4,5-j]phenanthridin-12-yl acetate
**3**	CO*n*Pr	(3S,4aS,11bS,12R)-3-methoxy-4,4a-dihydro-3H,6H-11b,5-ethano[1,3]dioxolo[4,5-j]phenanthridin-12-yl butyrate
**4**	CO*t*Bu	(3S,4aS,11bS,12R)-3-methoxy-4,4a-dihydro-3H,6H-11b,5-ethano[1,3]dioxolo[4,5-j]phenanthridin-12-yl pivalate
**5**	COC_5H_{11}	(3S,4aS,11bS,12R)-3-methoxy-4,4a-dihydro-3H,6H-11b,5-ethano[1,3]dioxolo[4,5-j]phenanthridin-12-yl hexanoate

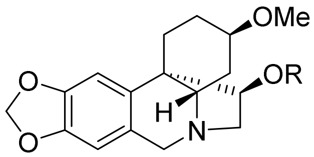

No	R	Name
**6**	H	(3R,4aS,11bS,12R)-3-methoxy-2,3,4,4a-tetrahydro-1H,6H-11b,5-ethano[1,3]dioxolo[4,5-j]phenanthridin-12-ol
**7**	Ac	(3R,4aS,11bS,12R)-3-methoxy-2,3,4,4a-tetrahydro-1H,6H-11b,5-ethano[1,3]dioxolo[4,5-j]phenanthridin-12-yl acetate
**8**	CO*n*Pr	(3R,4aS,11bS,12R)-3-methoxy-2,3,4,4a-tetrahydro-1H,6H-11b,5-ethano[1,3]dioxolo[4,5-j]phenanthridin-12-yl butyrate
**9**	CO*t*Bu	(3R,4aS,11bS,12R)-3-methoxy-2,3,4,4a-tetrahydro-1H,6H-11b,5-ethano[1,3]dioxolo[4,5-j]phenanthridin-12-yl pivalate
**10**	COC_5H_{11}	(3R,4aS,11bS,12R)-3-methoxy-2,3,4,4a-tetrahydro-1H,6H-11b,5-ethano[1,3]dioxolo[4,5-j]phenanthridin-12-yl hexanoate

Suplementary Figure S1. Structure of haemanthamine and dihydrohaemanthamine derivatives.

NMR data for compounds 1-10

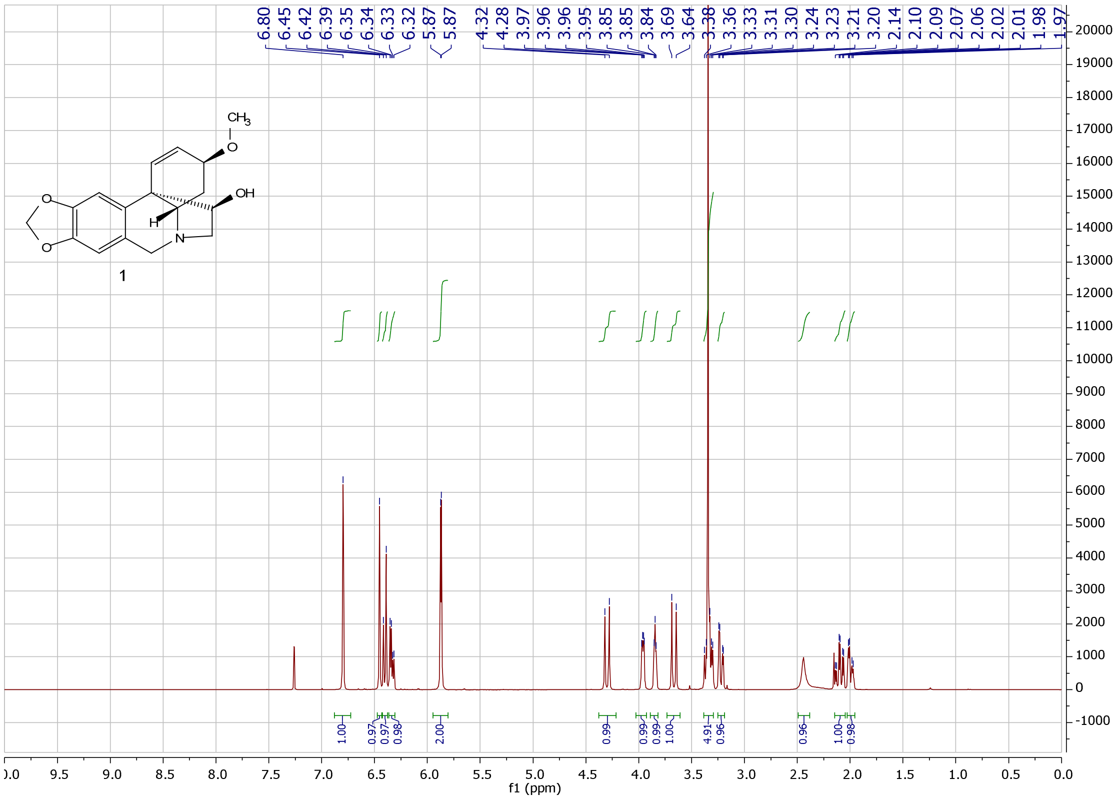

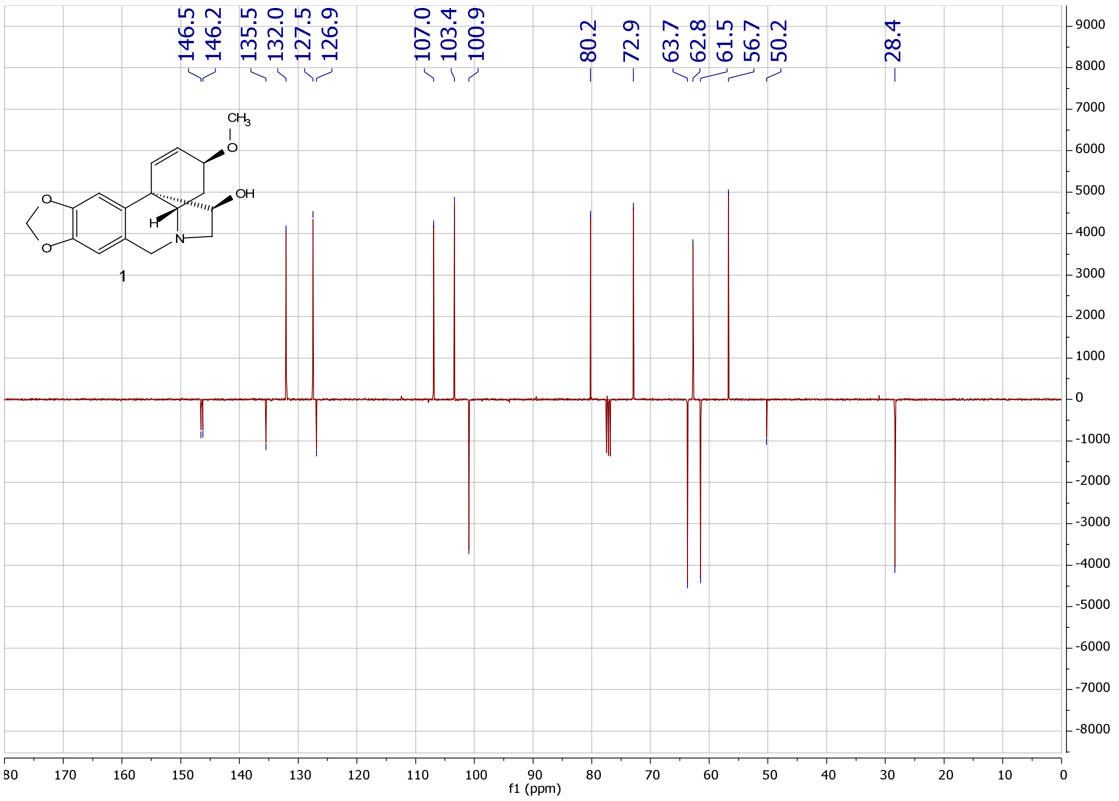

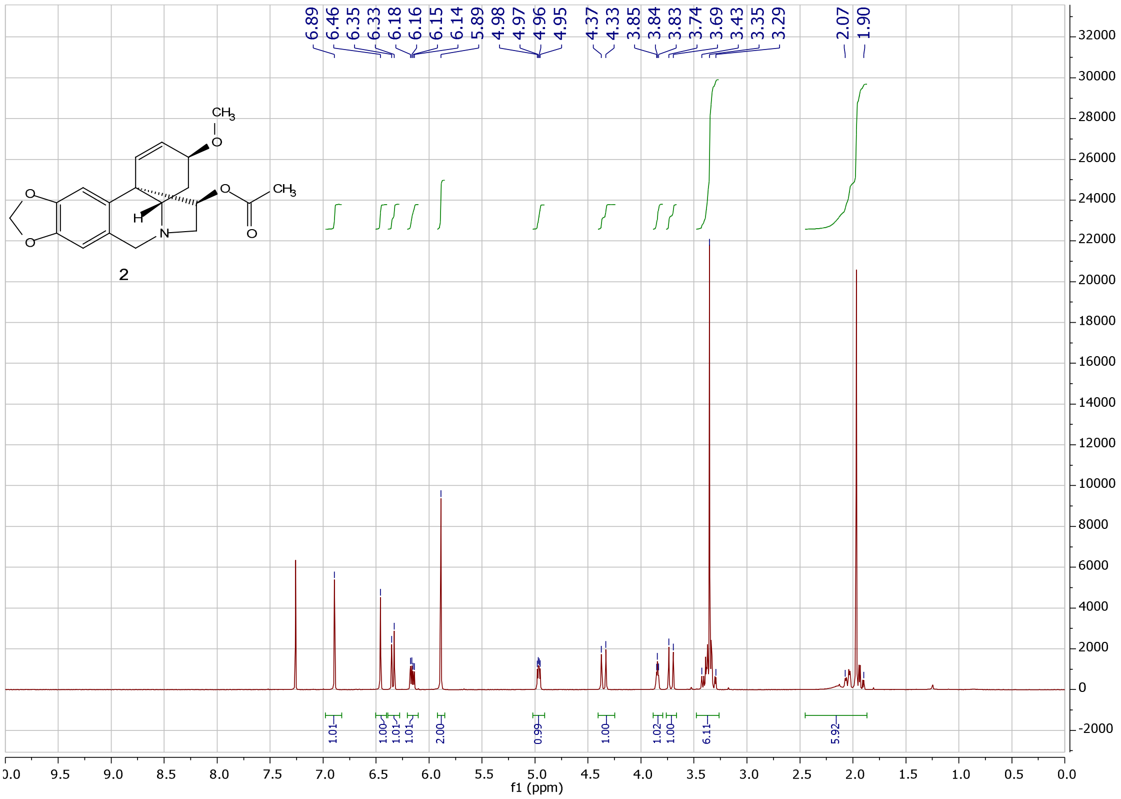

6.89
6.46
6.35
6.33
6.18
6.16
6.15
6.14
5.89
4.98
4.97
4.96
4.95
4.37
4.33
3.85
3.84
3.83
3.74
3.69
3.43
3.35
3.29
2.07
1.90
2
f1 (ppm)

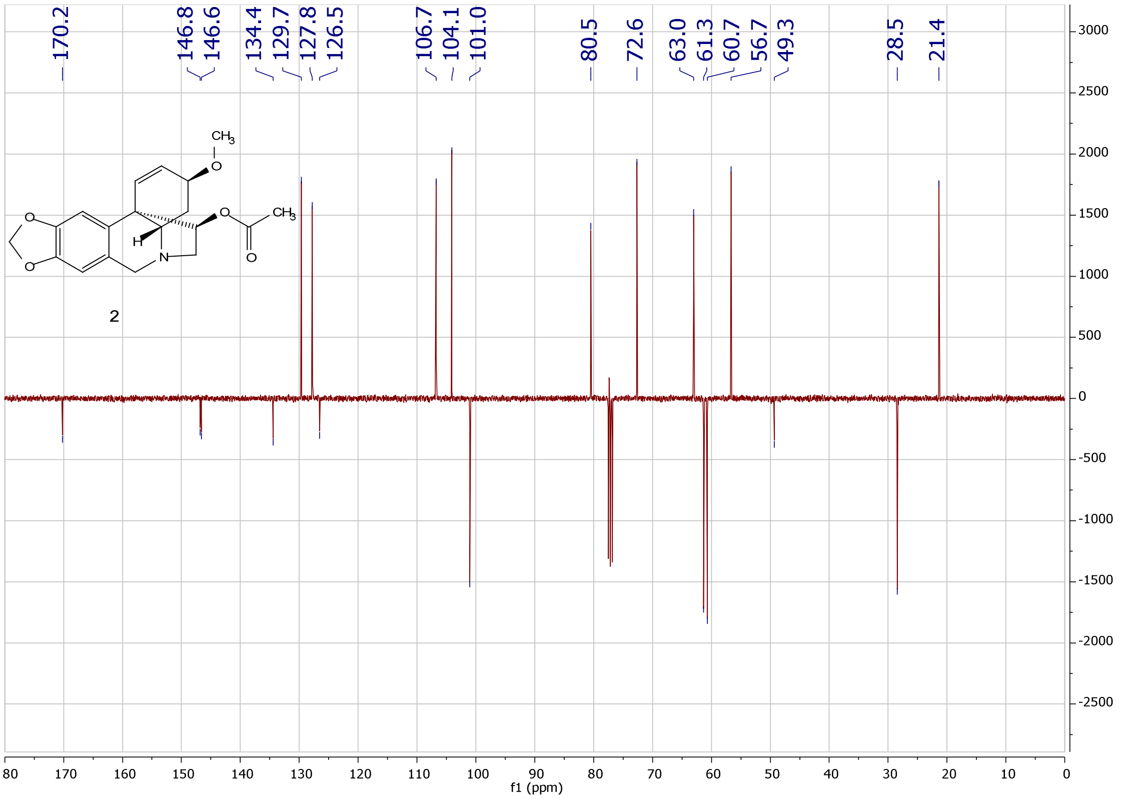

170.2
146.8
146.6
134.4
129.7
127.8
126.5
106.7
104.1
101.0
80.5
72.6
63.0
61.3
60.7
56.7
49.3
28.5
21.4
2
f1 (ppm)

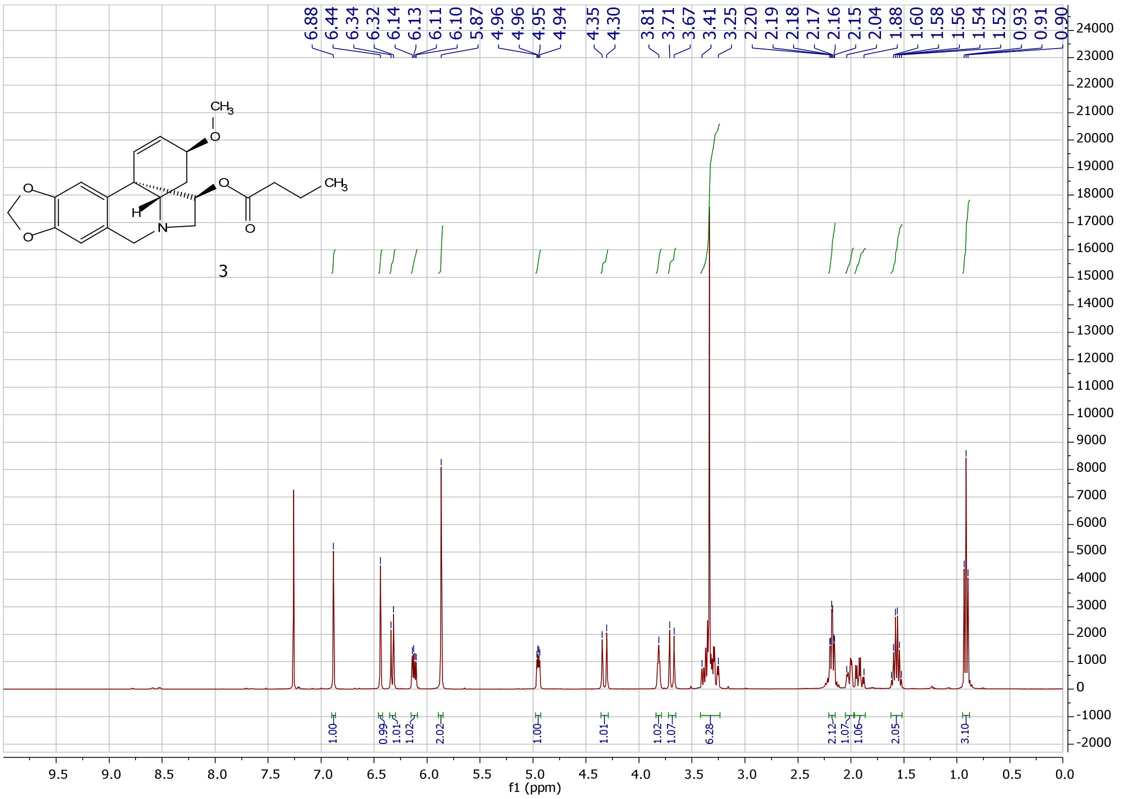
6.88
6.44
6.34
6.32
6.14
6.13
6.11
6.10
5.87
4.96
4.96
4.95
4.94
4.35
4.30
3.81
3.71
3.67
3.41
3.25
2.20
2.19
2.18
2.17
2.16
2.15
2.04
1.88
1.60
1.58
1.56
1.54
1.52
0.93
0.91
0.90
CH3
O
O
O
H
N
O
O
CH3
3
1.00
0.99
1.01
1.02
2.02
1.00
1.01
1.02
1.07
6.28
2.12
1.07
1.06
2.05
3.10
f1 (ppm)

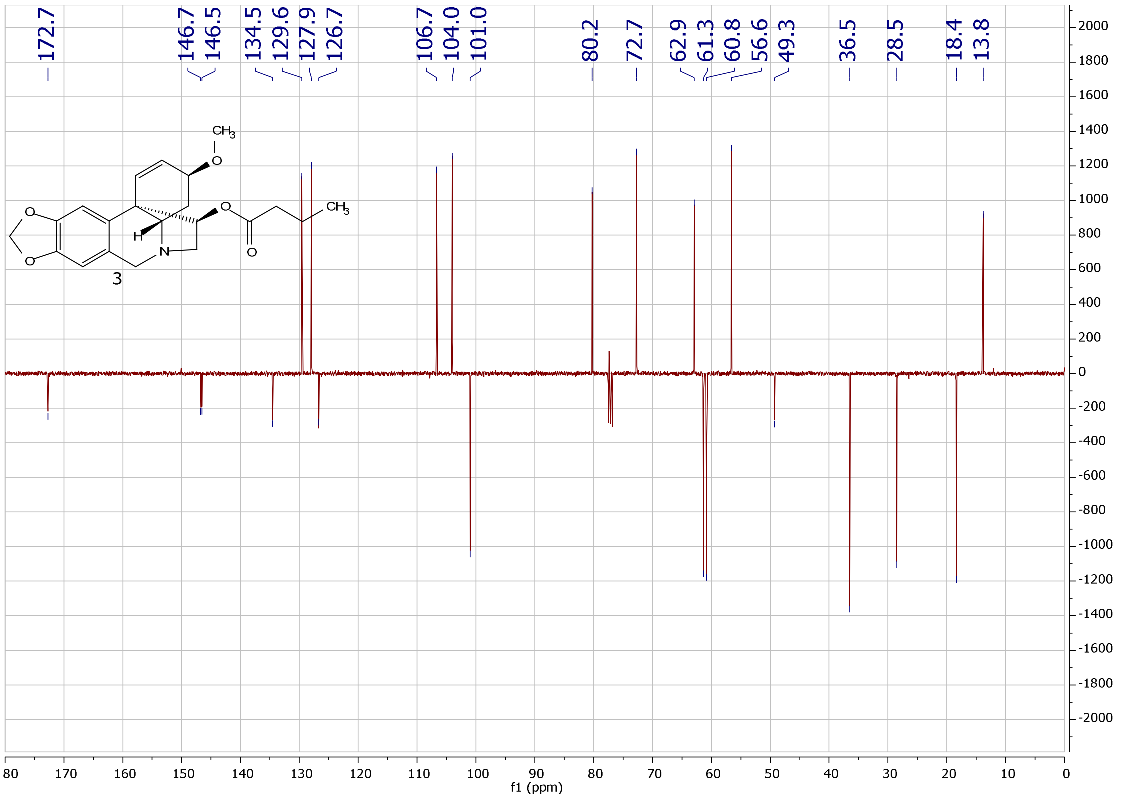
172.7
146.7
146.5
134.5
129.6
127.9
126.7
106.7
104.0
101.0
80.2
72.7
62.9
61.3
60.8
56.6
49.3
36.5
28.5
18.4
13.8
CH3
O
O
O
H
N
O
O
CH3
3
f1 (ppm)

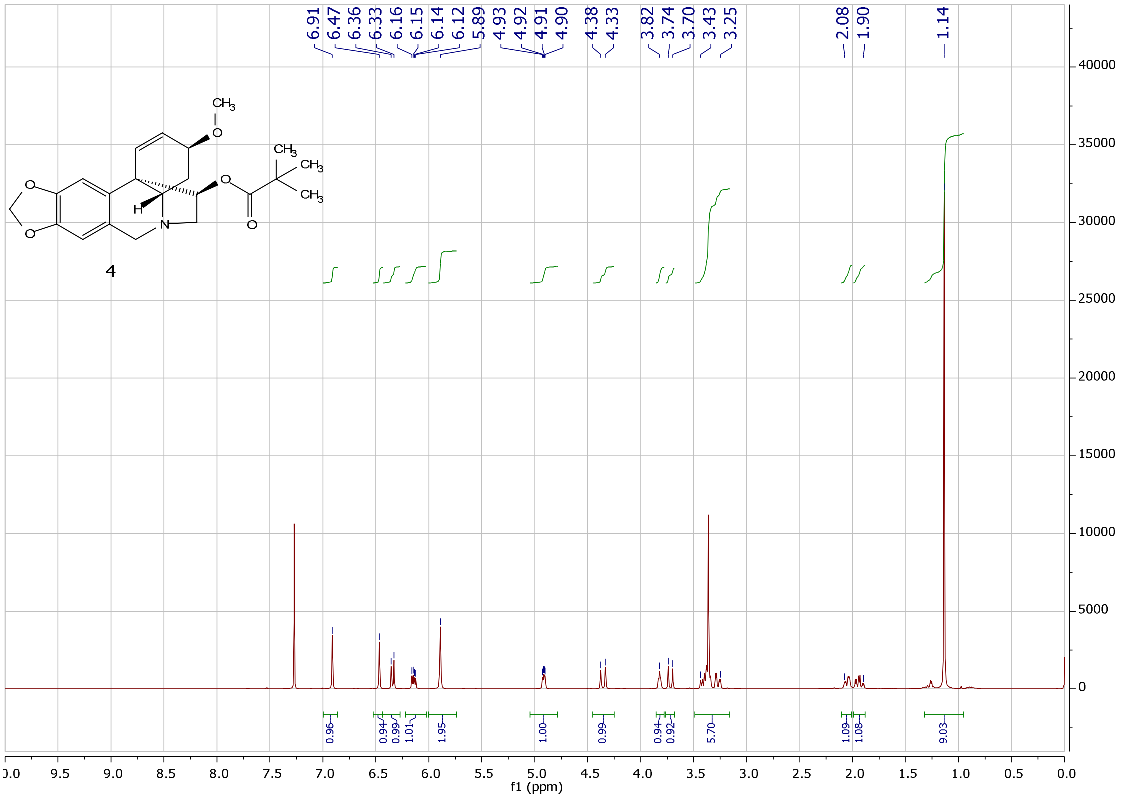

6.91
6.47
6.36
6.33
6.16
6.15
6.14
6.12
5.89
4.93
4.92
4.91
4.90
4.38
4.33
3.82
3.74
3.70
3.43
3.25
2.08
1.90
1.14
4
0.96
0.94
0.99
1.01
1.95
1.00
0.99
0.94
0.92
5.70
1.09
1.08
9.03
f1 (ppm)

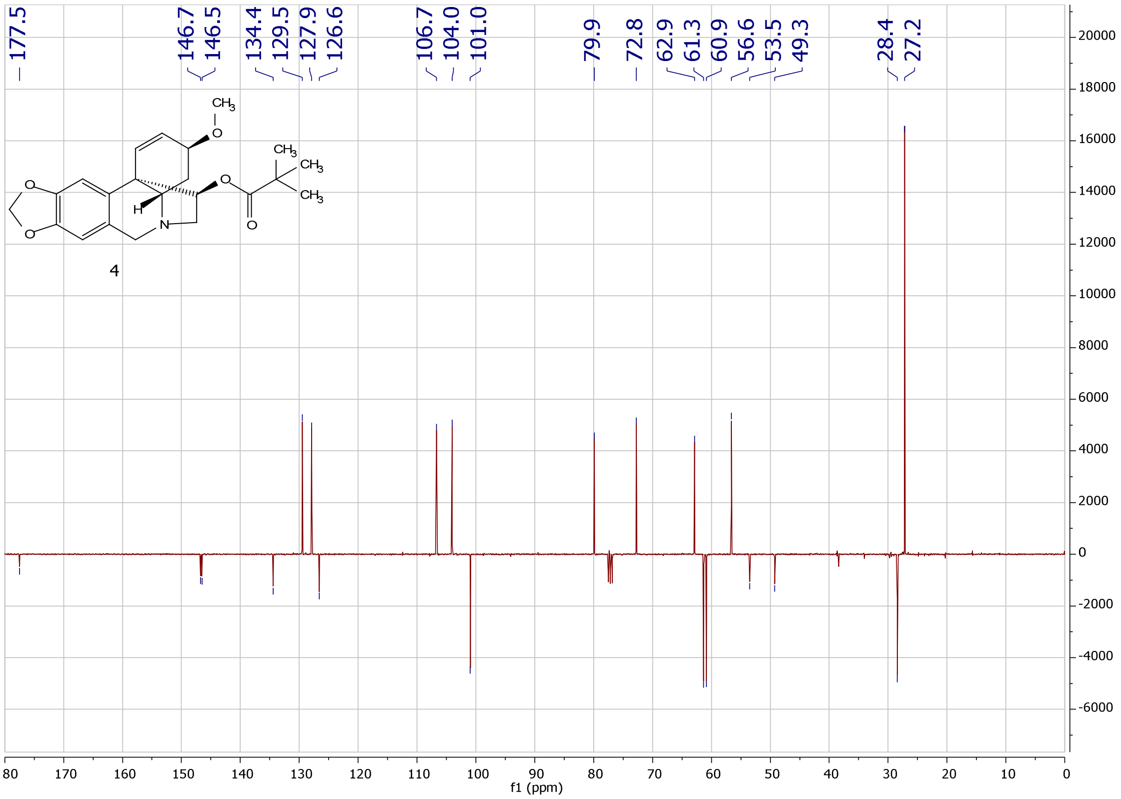

177.5
146.7
146.5
134.4
129.5
127.9
126.6
106.7
104.0
101.0
79.9
72.8
62.9
61.3
60.9
56.6
53.5
49.3
28.4
27.2
4
f1 (ppm)

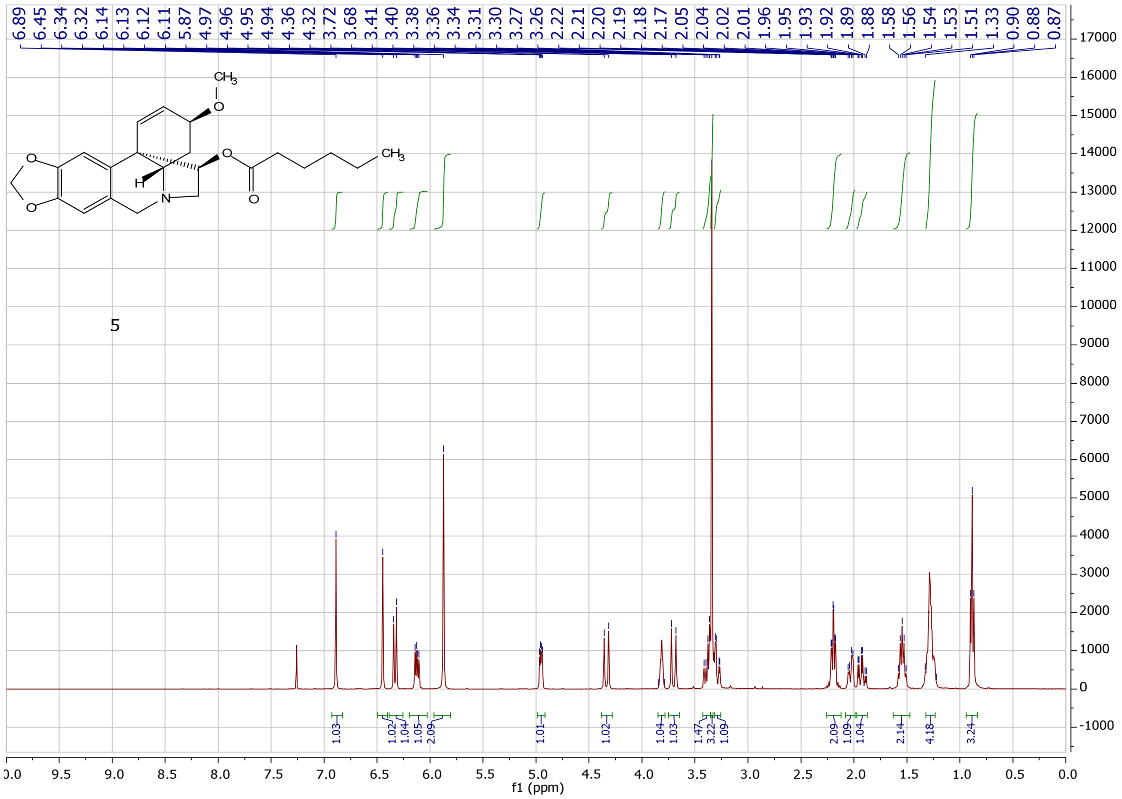
6.89 6.45 6.34 6.32 6.14 6.13 6.12 6.11 5.87 4.97 4.96 4.95 4.94 4.36 4.32 3.72 3.68 3.41 3.40 3.38 3.36 3.34 3.31 3.30 3.27 3.26 2.22 2.21 2.20 2.19 2.18 2.17 2.05 2.04 2.02 2.01 1.96 1.95 1.93 1.92 1.89 1.88 1.58 1.56 1.54 1.53 1.51 1.33 0.90 0.88 0.87
5
f1 (ppm)

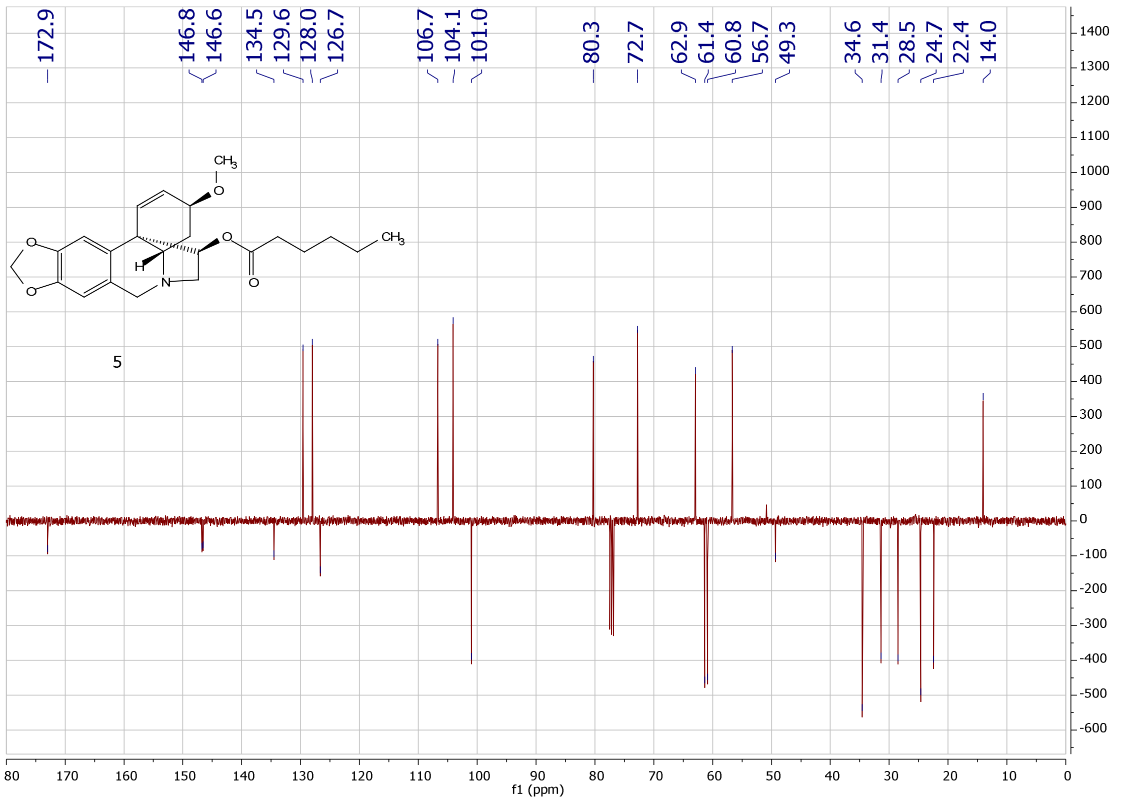
172.9 146.8 146.6 134.5 129.6 128.0 126.7 106.7 104.1 101.0 80.3 72.7 62.9 61.4 60.8 56.7 49.3 34.6 31.4 28.5 24.7 22.4 14.0
5
f1 (ppm)

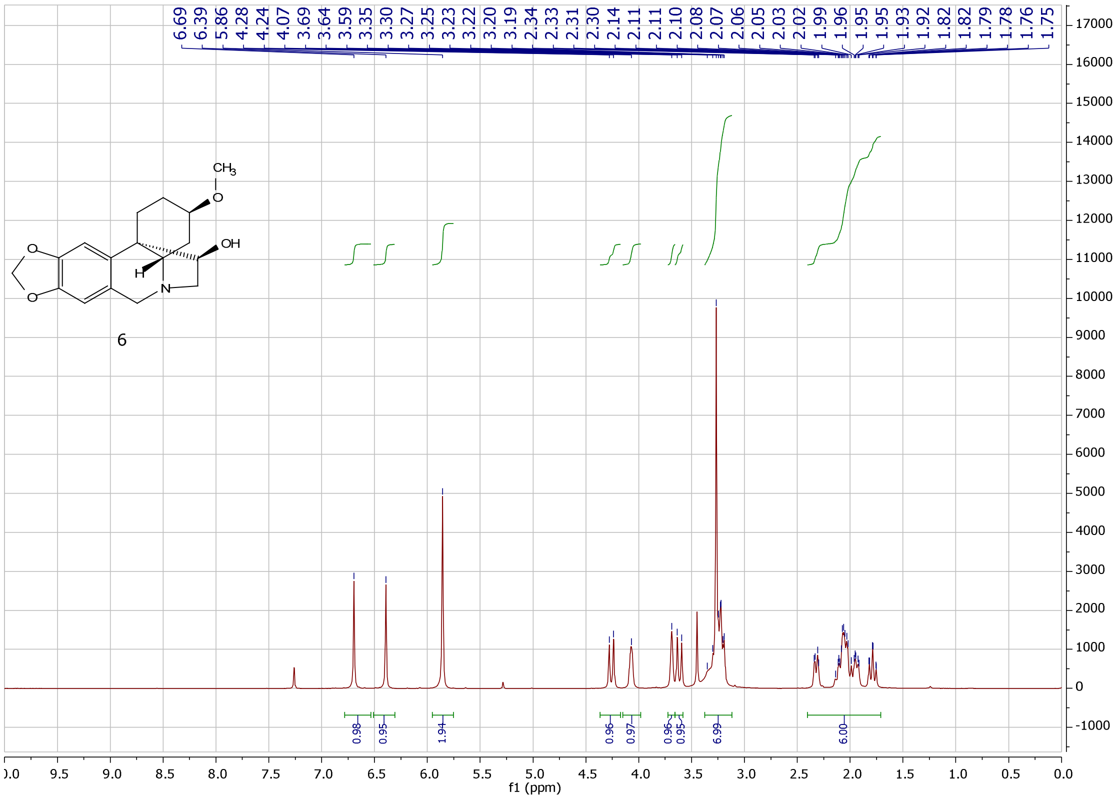
6.69 6.39 5.86 4.28 4.24 4.07 3.69 3.64 3.59 3.35 3.30 3.27 3.25 3.23 3.22 3.20 3.19 2.34 2.33 2.31 2.30 2.14 2.11 2.11 2.10 2.08 2.07 2.06 2.05 2.03 2.02 1.99 1.96 1.95 1.95 1.93 1.92 1.82 1.82 1.79 1.78 1.76 1.75
6
0.98 0.95 1.94 0.96 0.97 0.96 0.95 6.99 6.00
f1 (ppm)

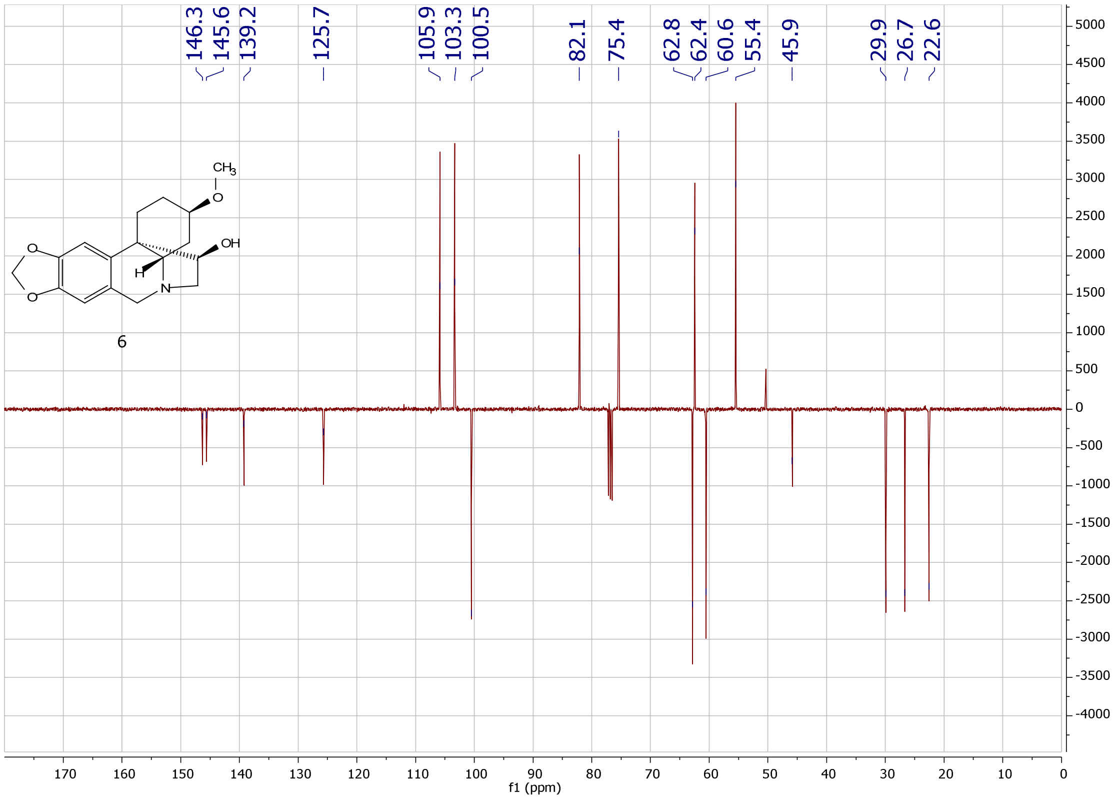
146.3 145.6 139.2 125.7 105.9 103.3 100.5 82.1 75.4 62.8 62.4 60.6 55.4 45.9 29.9 26.7 22.6
6
f1 (ppm)

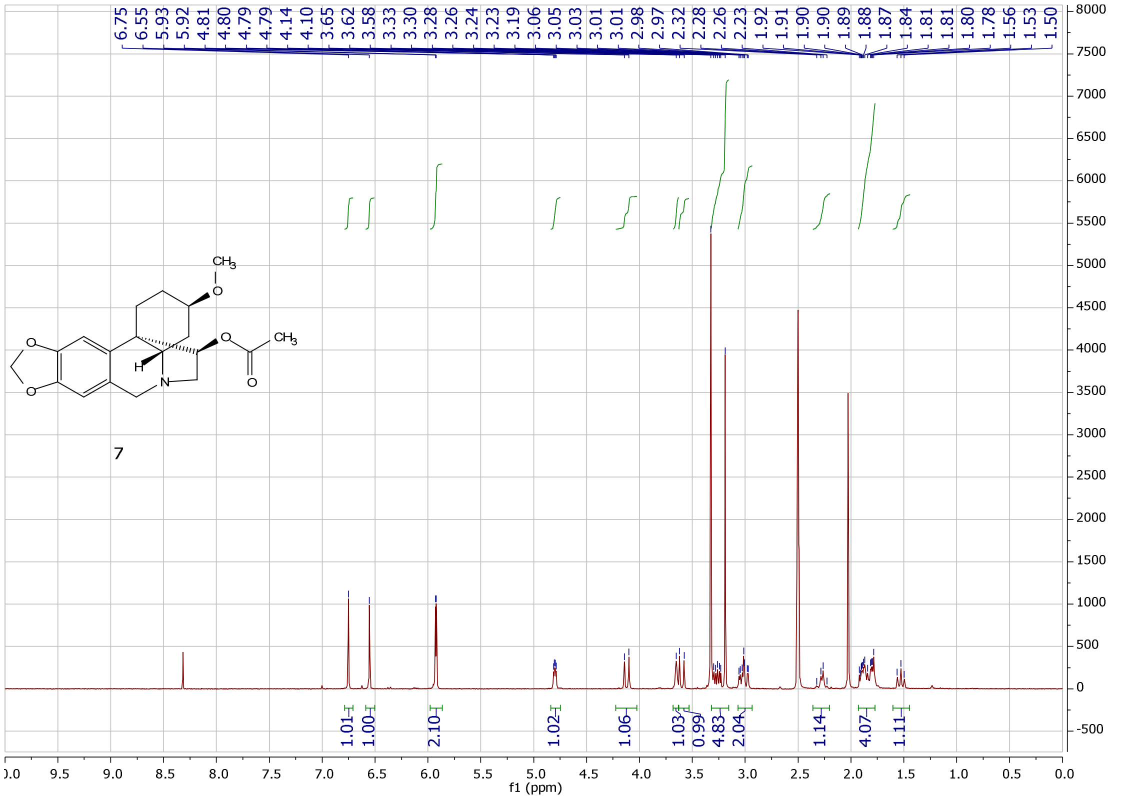

7

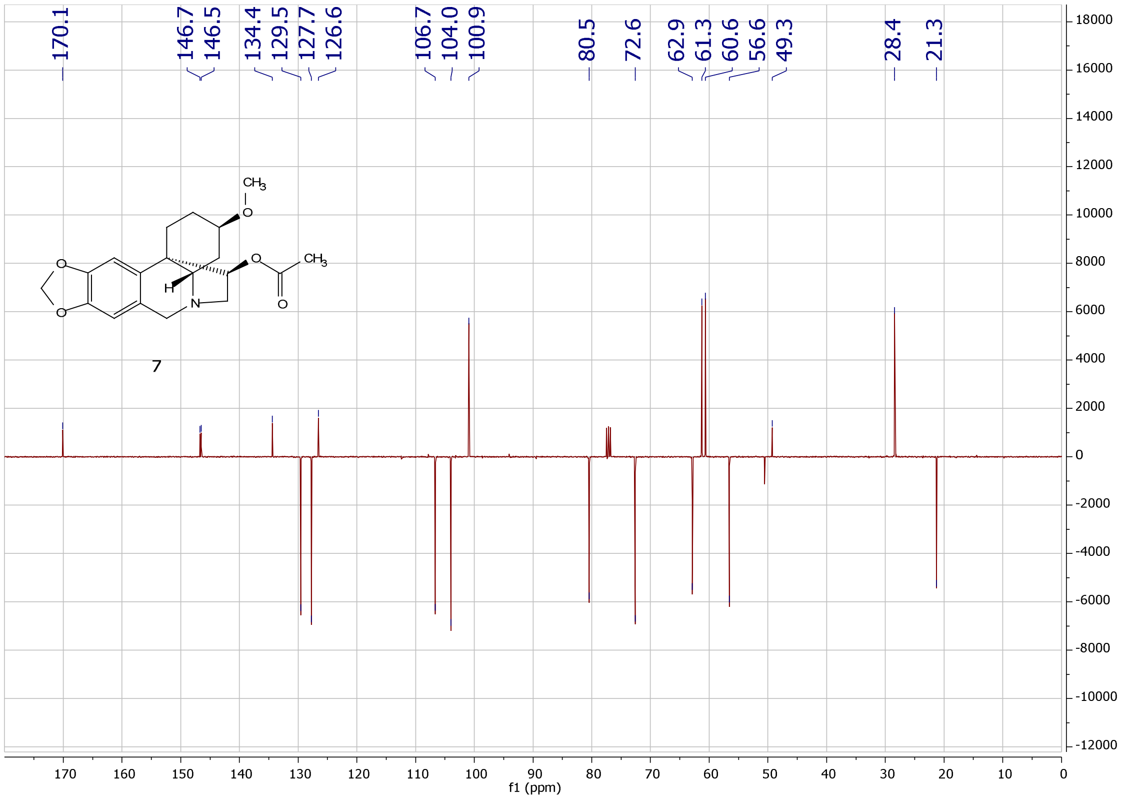

7

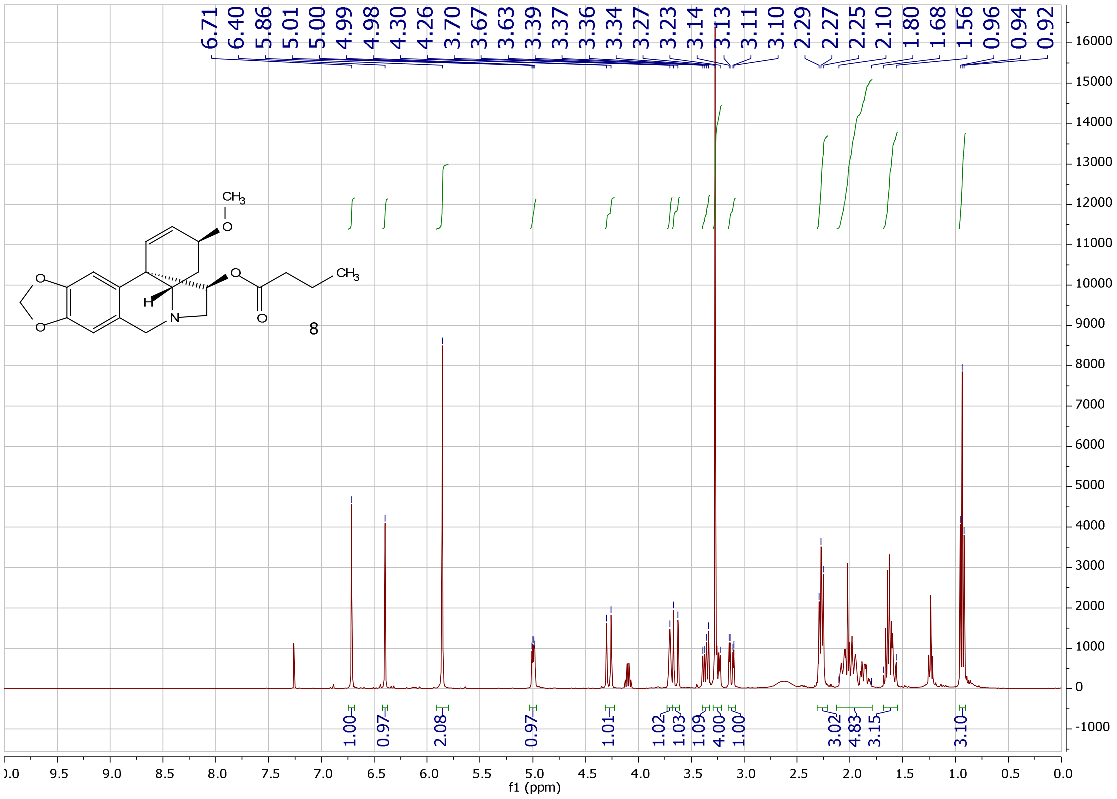
6.71 6.40 5.86 5.01 5.00 4.99 4.98 4.30 4.26 3.70 3.67 3.63 3.39 3.37 3.36 3.34 3.27 3.23 3.14 3.13 3.11 3.10 2.29 2.27 2.25 2.10 1.80 1.68 1.56 0.96 0.94 0.92
CH3
8
1.00 0.97 2.08 0.97 1.01 1.02 1.03 1.09 4.00 1.00 3.02 4.83 3.15 3.10
f1 (ppm)

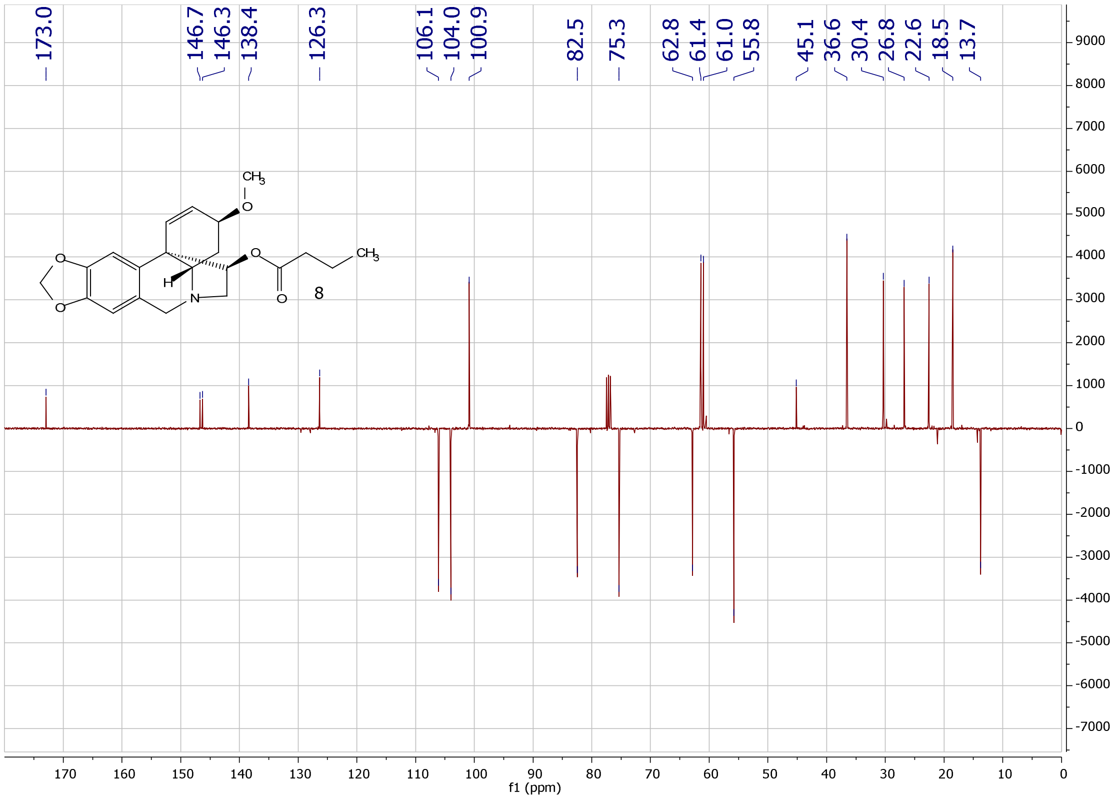
173.0 146.7 146.3 138.4 126.3 106.1 104.0 100.9 82.5 75.3 62.8 61.4 61.0 55.8 45.1 36.6 30.4 26.8 22.6 18.5 13.7
CH3
8
f1 (ppm)

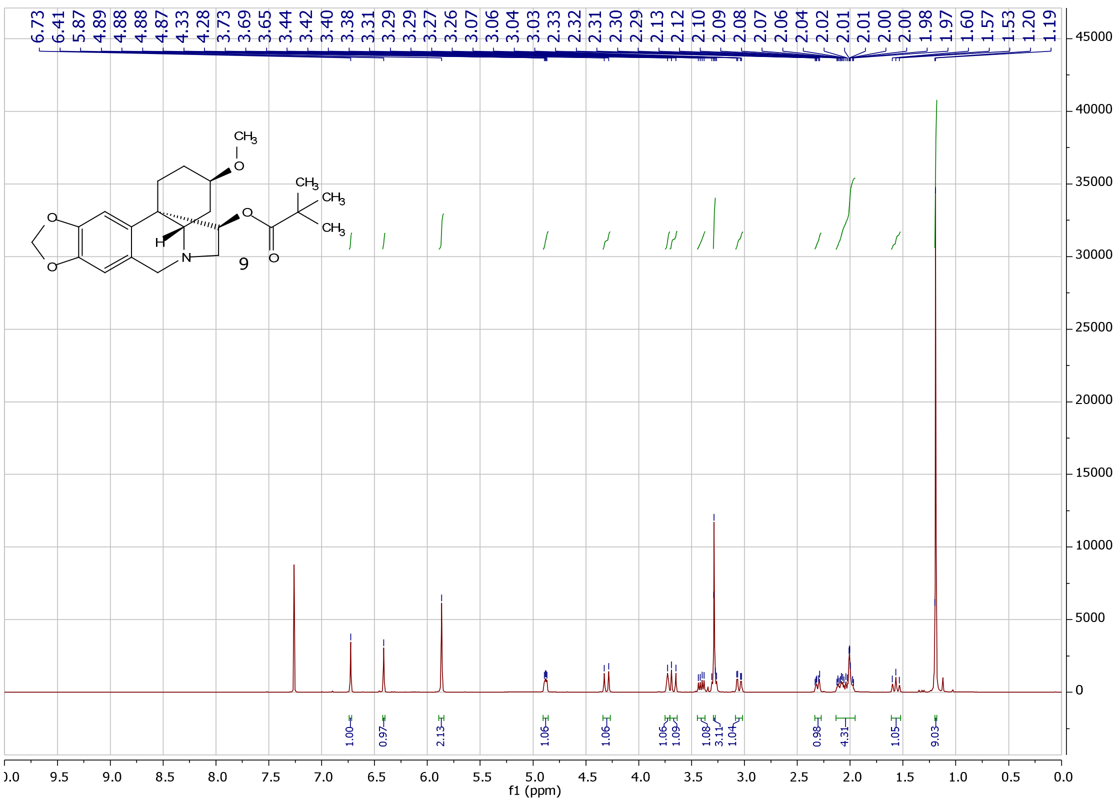
6.73 6.41 5.87 4.89 4.88 4.88 4.87 4.33 4.28 3.73 3.69 3.65 3.44 3.42 3.40 3.38 3.31 3.29 3.29 3.27 3.26 3.07 3.06 3.04 3.03 2.33 2.32 2.31 2.30 2.29 2.13 2.12 2.10 2.09 2.08 2.07 2.06 2.04 2.02 2.01 2.01 2.00 2.00 1.98 1.97 1.60 1.57 1.53 1.20 1.19
1.00 0.97 2.13 1.06 1.06 1.06 1.09 1.08 3.11 1.04 0.98 4.31 1.05 9.03
CH3
O
CH3
CH3
CH3
O
O
H
N
O
O
9
f1 (ppm)

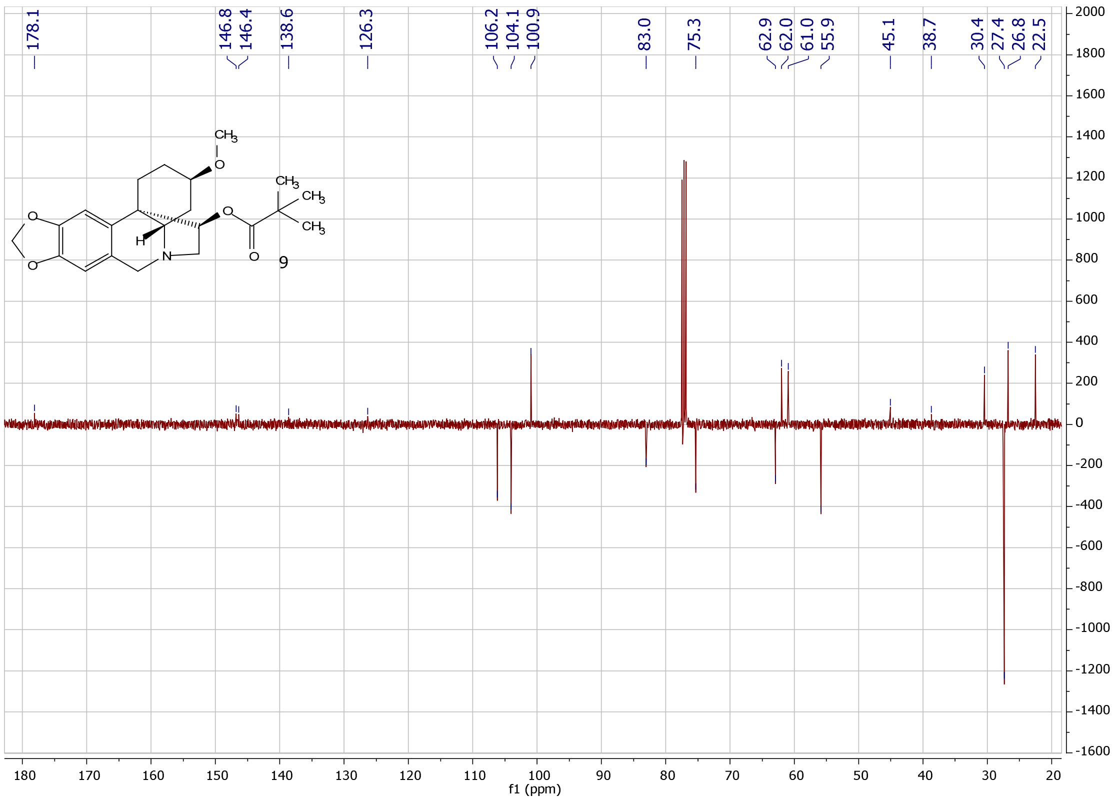
178.1 146.8 146.4 138.6 126.3 106.2 104.1 100.9 83.0 75.3 62.9 62.0 61.0 55.9 45.1 38.7 30.4 27.4 26.8 22.5
CH3
O
CH3
CH3
CH3
O
O
H
N
O
O
9
f1 (ppm)

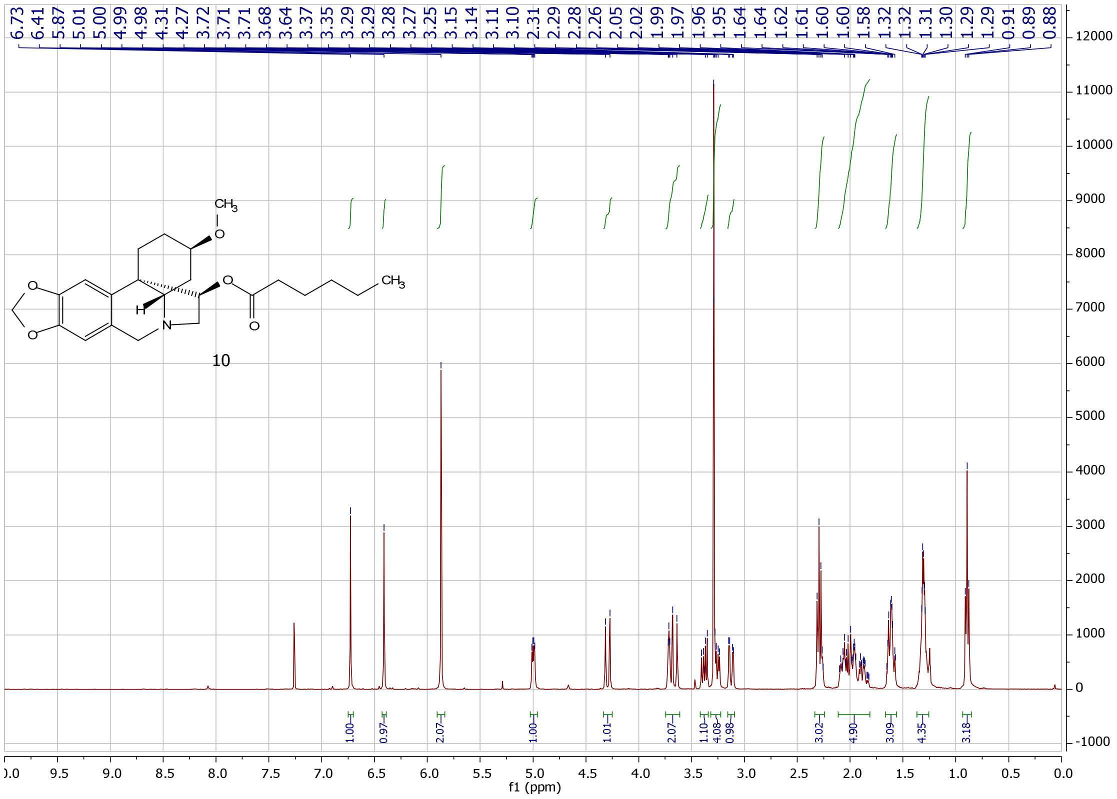
6.73
6.41
5.87
5.01
5.00
4.99
4.98
4.31
4.27
3.72
3.71
3.71
3.68
3.64
3.37
3.35
3.29
3.29
3.28
3.27
3.25
3.15
3.14
3.11
3.10
2.31
2.29
2.28
2.26
2.05
2.02
1.99
1.97
1.96
1.95
1.64
1.62
1.61
1.60
1.60
1.58
1.32
1.32
1.31
1.30
1.29
1.29
0.91
0.89
0.88
CH3
O
O
O
H
N
O
O
CH3
10
1.00
0.97
2.07
1.00
1.01
2.07
1.10
4.08
0.98
3.02
4.90
3.09
4.35
3.18
f1 (ppm)

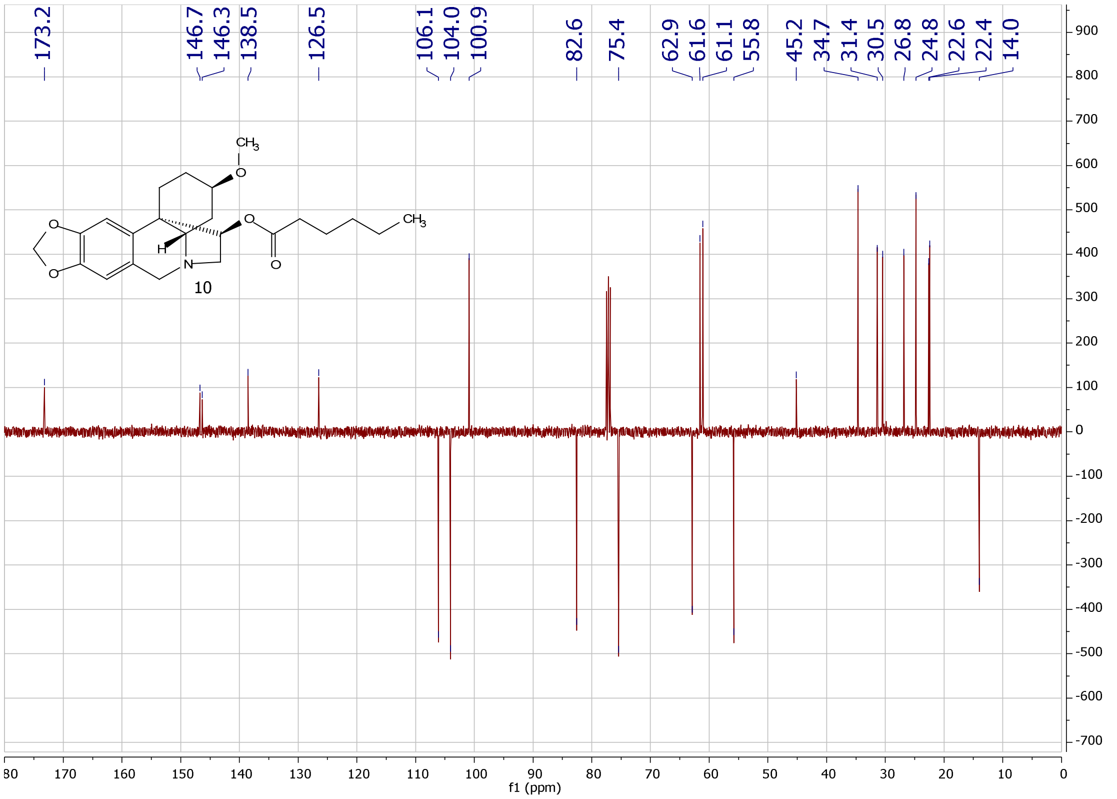
173.2
146.7
146.3
138.5
126.5
106.1
104.0
100.9
82.6
75.4
62.9
61.6
61.1
55.8
45.2
34.7
31.4
30.5
26.8
24.8
22.6
22.4
14.0
CH3
O
O
O
H
N
O
O
CH3
10
f1 (ppm)

Filename: SI proofs
Directory: H:
Template: C:\Users\eramos-morales\AppData\Local\Microsoft\Windows\Temporary Internet Files\Content.MSO\7E4C691B.dotm
Title:
Subject:
Author: Eva
Keywords:
Comments:
Creation Date: 18/01/2019 12:59:00
Change Number: 3
Last Saved On: 18/01/2019 12:59:00
Last Saved By: Eva Ramos-Morales
Total Editing Time: 2 Minutes
Last Printed On: 18/01/2019 15:31:00
As of Last Complete Printing
Number of Pages: 17
Number of Words: 2,504 (approx.)
Number of Characters: 14,275 (approx.)
